# Historical effective population size of North American hoary bat (*Lasiurus cinereus*) and challenges to estimating trends in contemporary effective breeding population size from archived samples

**DOI:** 10.7717/peerj.11285

**Published:** 2021-04-19

**Authors:** Robert S. Cornman, Jennifer A. Fike, Sara J. Oyler-McCance, Paul M. Cryan

**Affiliations:** U.S. Geological Survey, Fort Collins Science Center, Fort Collins, CO, United States of America

**Keywords:** Hoary bat, Effective population size, Population genetics, Genome sequencing, Reduced representation sequencing, Wind turbine mortality

## Abstract

**Background:**

Hoary bats (*Lasiurus cinereus*) are among the bat species most commonly killed by wind turbine strikes in the midwestern United States. The impact of this mortality on species census size is not understood, due in part to the difficulty of estimating population size for this highly migratory and elusive species. Genetic effective population size (Ne) could provide an index of changing census population size if other factors affecting Ne are stable.

**Methods:**

We used the NeEstimator package to derive effective breeding population size (Nb) estimates for two temporally spaced cohorts: 93 hoary bats collected in 2009–2010 and an additional 93 collected in 2017–2018. We sequenced restriction-site associated polymorphisms and generated a de novo genome assembly to guide the removal of sex-linked and multi-copy loci, as well as identify physically linked markers.

**Results:**

Analysis of the reference genome with *psmc* suggested at least a doubling of Ne in the last 100,000 years, likely exceeding Ne = 10,000 in the Holocene. Allele and genotype frequency analyses confirmed that the two cohorts were comparable, although some samples had unusually high or low observed heterozygosities. Additionally, the older cohort had lower mean coverage and greater variability in coverage, and batch effects of sampling locality were observed that were consistent with sample degradation. We therefore excluded samples with low coverage or outlier heterozygosity, as well as loci with sequence coverage far from the mode value, from the final data set. Prior to excluding these outliers, contemporary Nb estimates were significantly higher in the more recent cohort, but this finding was driven by high values for the 2018 sample year and low values for all other years. In the reduced data set, Nb did not differ significantly between cohorts. We found base substitutions to be strongly biased toward cytosine to thymine or the complement, and further partitioning loci by substitution type had a strong effect on Nb estimates. Minor allele frequency and base quality bias thresholds also had strong effects on Nb estimates. Instability of Nb with respect to common data filtering parameters and empirically identified factors prevented robust comparison of the two cohorts. Given that confidence intervals frequently included infinity as the stringency of data filtering increased, contemporary trends in Nb of North American hoary bats may not be tractable with the linkage disequilibrium method, at least using the protocol employed here.

## Introduction

Bats provide valuable ecological services in the form of pollination, seed dispersal, and predation of pest insects, yet are under-studied as a group and many species are under threat (Voigt & Kingston, 2016). In the United States, some bat populations face new and potentially existential threats from white-nose syndrome (Frick et al., 2010) and industrial wind turbines (Kunz et al., 2007; Cryan, 2011; O’Shea et al., 2016; Frick et al., 2017). While the scale of bat mortality associated with wind energy development is clearly large relative to plausible estimates of reproductive rates (Frick et al., 2017), the most susceptible taxa are among the most poorly censused. Hoary bats (*Lasiurus [Aeroestes] cinereus*) comprise at least a third of annual bat fatalities occurring at wind turbines in the United States and Canada (Smallwood, 2013; Thompson et al., 2017). This highly migratory species is commonly found in late summer and autumn at most wind facilities where carcass monitoring occurs, disproportionately so relative to regional bat diversity (Arnett & Baerwald, 2013). Infrared imaging has revealed a strong behavioral component of turbine strikes, in which individuals repeatedly approach wind turbine surfaces and moving blades (Horn, Arnett & Kunz, 2008; Cryan et al., 2014a). While fatality reduction strategies are being developed (e.g., Smallwood & Bell, 2020; Weaver et al., 2020), there is an urgent need to understand population trends concurrent with wind energy development, as our limited understanding can exclude neither extinction nor stable co-existence as eventual outcomes (Frick et al., 2017).

Censusing hoary bat populations is challenging not only for their nocturnal behavior and dispersed roosting in trees but also their extreme migratory habits juxtaposed with long periods of inactivity in cryptic hibernation sites (Kunz & Parsons, 2009; O’Shea & Bogan, 2003). Tagged individuals have been found to travel thousands of linear kilometers in short periods (Weller et al., 2016), and males and females live apart in disparate geographic regions for much of the year (Findley & Jones, 1964; Hayes, Cryan & Wunder, 2015). Given these challenges, a number of studies have employed genetic methods to illuminate past demographic history and current genetic structure of hoary bat populations (Korstian, Hale & Williams, 2015; Russell et al., 2015; Sovic, Carstens & Gibbs, 2016; Pylant et al., 2016). Several studies included estimates of genetic effective population size (Ne), a theoretical parameter of population-genetic models that describes how life-history traits affect the sampling variance of allele frequencies. Ne originates from the classic work of Sewall Wright and is conceptualized as the size of an ideal random-mating population that would have the same sampling variance (i.e., genetic drift) as the real population of interest, and at model equilibrium is less than the census population size (Nc) due to various biological sources of reproductive variance. From an appropriate sample of genetic variation, the population Ne can be estimated and the effects of predictor variables empirically evaluated (Charlesworth, 2009).

Various approaches to Ne estimation have been proposed, which are differentially impacted by nonequilibrium conditions in the biological populations to which they are applied. Some estimators specify particular components of allele sampling variance such as due to inbreeding or the breeding population size (Nb) (Wang, Santiago & Caballero, 2016). Methods are further distinguished by the extent to which the estimate is ‘contemporary’ or compounds effects of past generations. Comparing Ne estimated by different methodologies can therefore be fraught and for most methods and realistic sample sizes, confidence intervals are often large or unbounded (Do et al., 2014). Estimating contemporary trends in Ne is particularly challenging, as large samples must be genotyped at numerous loci and over a time interval sufficient to reveal informative genetic change (Tallmon et al., 2012; Luikart et al., 2020). Fortunately, continued gains in sequencing methodology and throughput have advanced the prospects of contemporary Ne estimation. Reduced-representation sequencing is efficient for genotyping thousands of variant sites for dozens of individuals, particularly using restriction digest protocols (“RADseq”) (Peterson et al., 2012). Here we apply this technology to investigate temporal trends in contemporary Nb in hoary bats, as a proxy for temporal change in Ne generally or Nc.

Two disjunct American populations of hoary bats of unknown divergence time are presumed to be genetically isolated in the present day: a North American population spanning much of North America into southern Mexico, and a disjunct South American population (Shump Jr & Shump, 1982; Cryan, 2003; Baird et al., 2015; Russell et al., 2015). For this analysis, we assume these two continental populations are independent with respect to contemporary Ne but not ancestral Ne. *L. cinereus* has also colonized archipelagos such as the Hawaiian and Galapagos Islands (Koopman & McCracken, 1998; Baird et al., 2015; Russell et al., 2015; Ziegler, Howarth & Simmons, 2016). Our focus here is on hoary bats that migrate through the central United States, which we presume represent the majority of the North American population and also the group most impacted by turbine strikes (Cryan, Stricker & Wunder, 2014b). In this work, we retain the genus classification of *Lasiurus* following Novaes et al. (2018), although this placement is not material to our results.

Previous genetic studies indicate that the North American population of hoary bat is unstructured (Korstian, Hale & Williams, 2015; Pylant et al., 2016; Sovic, Carstens & Gibbs, 2016), but also suggest a large plausible interval for contemporary Ne or Nb. The latter is important because the dynamic range over which Ne estimators are useful varies (Do et al., 2014; Wang, 2016). For example, Sovic, Carstens & Gibbs (2016) previously estimated a large Ne for *L. cinereus*, approximately 8.3E+5 based on coalescent modeling of site frequency spectra of single nucleotide polymorphisms (SNPs) and in the context of an explicit model of recent population growth. This value was intermediate between those of its congeners the silver-haired bat (*L. noctivagans*, Ne = 1.9E+5) and the eastern red bat (*L. borealis*, Ne = 1.6E+6). Ne estimates modeled by Pylant et al. (2016) from mitochondrial and microsatellite data were comparable to Sovic et al. for *L. borealis* (3.6E+6) but were more than an order of magnitude lower for *L. cinereus* (6.1E+4). In contrast, Korstian, Hale & Williams (2015) could not resolve Nb for *L. cinereus*, using the linkage disequilibrium (LD) method as modified by Waples & Do (2010). They obtained negative estimates with microsatellite markers, which implies that the scale of the sampling correction implemented by that method was greater than the effect of Nb itself, i.e., that *L. cinereus* Nb was too large to estimate. On the other hand, those authors estimated a Nb of only 1,546 from a large sample of *L. borealis*.

To investigate temporal trends in Nb, we used archived samples with temporal breadth likely exceeding the generation time of *L. cinereus*, defined as the expected time between the birth of a female and the birth of her first female progeny (Charlesworth, 1994) and usually much less than expected lifespan. We performed RADseq genotyping for two temporal cohorts initially consisting of 93 individuals, one set of caracasses collected from 2009–2010 and a second set from 2017–2018, a mean difference of eight years. This strategy maximized the available size and balance of each cohort, with size limited primarily at the earlier time period, without making individual cohorts overly broad. This temporal scale is certainly less than the 5–10 generations estimated by Tallmon et al. (2010) and Tallmon et al. (2012) to be required to detect a 10% shift from a stable growth parameter (*λ* = 1). More recently, Luikart et al. (2020) found that with 5,000 SNPs the LD method could detect differences in Nb on the order of 25% when the true value was on the order of hundreds, but with larger samples than used here. Thus, our analysis may have relatively low power to detect trends in Nb at this time scale, but a proactive strategy is prudent and there remains a need to establish a clearer baseline for future genetic monitoring (Schwartz, Luikart & Waples, 2007). For example, Luikart et al. (2020) established a framework for inferring Nb trends by linear regression over a time series, to which the current work could eventually be adapted.

Nb was estimated from biallelic SNPs using the LD method, which has been shown to be among the most accurate and robust of available methods for estimating contemporary Nb (Do et al., 2014; Wang, 2016). Several additional considerations guided our use of the LD method to estimate Nb, the first of which is its wide dynamic range. While Nb estimates are more precise at lower values of true Nb (Waples & Do, 2010) and simulations of pre-genomic data sets indicated that Nb above 1,000 were not easily differentiable (estimates were often infinite Waples & Do, 2010), Wang (2016) showed that typical genomic SNP datasets could achieve accurate estimation for true Nb well above 10,000. The LD method is also only slightly biased by large, sudden changes in Nb (Wang, 2016), which might potentially apply to *L. cinereus* given current turbine-strike estimates (Frick et al., 2017). While the LD method effectively integrates Nb across preceding generations in a weighted fashion, the most recent generation should predominate if mating is random and generations are discrete and non-overlapping (Waples, 2005; Wang, 2016). Random mating is suggested by an overall lack of genetic structure in *L. cinereus* (Korstian, Hale & Williams, 2015; Pylant et al., 2016; Sovic, Carstens & Gibbs, 2016) and no direct evidence of nonrandom mating has been reported to our knowledge. However, the assumption that generations do not overlap is probably universally violated in bats. We must therefore assume that opportunistic samples of sufficient size have convergent demographic structure and therefore deviate from the second assumption to a similar degree. Thus, while we refer to estimates as Nb, we assume they reflect the temporal mean of Ne and Nb of the overlapping generations within each cohort based on the results of Waples, Antao & Luikart (2014). Such estimates tend to be downwardly biased due to a temporal Wahlund effect (Waples, Antao & Luikart, 2014).

The LD method assumes marker loci are not physically linked, i.e., they are on separate chromosomes or effectively unlinked due to recombination. Failure to account for physical linkage with large SNP data sets substantially underestimates true Nb (Wang, 2016) and causes confidence intervals to be estimated too narrowly (Waples, Larson & Waples, 2016). We therefore assembled a reference genome to place anonymous loci onto genomic scaffolds. The reference genome also aided the detection of sex-linked and multicopy loci, and was achieved at relatively low cost with current scaffolding technologies. Note, the number of chromosomes inherently limits the LD method regardless of whether loci are anonymous or not, but this limitation was tangible only for very small chromosome numbers (approximately four or less) in simulations by Waples, Larson & Waples (2016), whereas the haploid chromosome number of *L. cinereus* is 14 (Baker & Patton, 1967). Waples, Larson & Waples (2016) also found a relatively modest benefit of 4,000 loci over 1,000 loci, suggesting that quality may be preferred over quantity when many thousands of SNP loci have been ascertained.

While we found data processing to have a strong effect on Nb, estimates were initially higher in the 2017–2018 cohort than the 2009–2010 cohort. However, removal of loci with outlier coverage patterns and samples with outlier observed heterozygosity (H_o_) largely eliminated the apparent difference between cohorts. Furthermore, we identified effects attributable to sample condition that limited interpretation of our results. We found that the most stringent filtering of samples, loci, and SNP genotyping parameters produced unbounded Nb estimates, which may ultimately preclude trend analysis for hoary bat Nb even if issues related to sample condition are resolved.

## Materials & Methods

### Sampling

Samples consisted of carcasses found dead at wind turbines, aggregated from diverse monitoring activities, and taxonomically verified, labeled, and stored by the US Fish and Wildlife Service (USFWS). Samples were stored frozen in chest freezers without defrost or thaw cycles, although the operational temperature was not recorded. All samples were collected from wind-energy sites in Indiana merely due to USFWS administrative boundaries, which has no expected bearing on study outcomes. After reviewing the number of samples available by collection year, two cohorts were identified that were sufficiently represented for analysis, 2009–2010 and 2017–2018. Two-year cohorts were chosen in order to maximize both sample size and sample balance. Samples within two-year cohorts are presumed to be separated by substantially less than a generation whereas the period of seven to nine years between cohorts is presumed to be substantially greater than the generation time of the species based on data for bats generally, as we are not aware of explicit generation time estimates for hoary bat. Hoary bats become sexually mature in their first year and reproductive females typically bear two pups per litter (range 1–4; Shump Jr & Shump, 1982; Cryan et al., 2012), investing a large amount of available energy in offspring (Racey & Entwistle, 2000). On the other hand, maximum lifespan of *L. cinereus* could plausibly exceed ten years (Wilkinson & South, 2002) and early onset of sexual maturity does not necessarily entail early reproductive success. On balance, a generation time of 2–4 years seems reasonable to assume given current understanding of reproductive phenology in this species (Cryan et al., 2012).

Samples from 2010, 2017, and 2018 were assigned distinct sample codes by the USFWS to designate batches obtained from specific localities (File S1), which have been anonymized here for privacy. As sample condition at discovery may have varied systematically by batch based on the frequency of surveys at each site, or variable storage or transit conditions prior to receipt by the USFWS, we investigated whether batch effects were evident among these sets (see ‘Results’). We excluded 2009 samples from single-year analysis because relatively few were available and site codes had not been assigned at that point.

### DNA extraction

Wing tissue clips were removed from frozen samples by the USFWS and shipped in 95% ethanol to the Molecular Ecology Laboratory, US Geological Survey (USGS), Fort Collins Science Center. Genomic DNA was extracted from wing tissue using an ammonium acetate protocol modified from the Gentra PureGene kit (Qiagen Inc.) for use with small amounts of tissue and solutions made in house. Genomic DNA was quantified using a broad range dsDNA assay kit on a qubit fluorometer (Life Technologies).

Tissue for genome sequencing was obtained from an adult female *L. cinereus* opportunistically collected through a regional public health organization in Fort Collins, Colorado on 9 September 2019. Blood was drawn into an EDTA blood collection tube within 12 h *post mortem* and cold-shipped to MedGenome, Inc. for specialized extraction of high-molecular weight genomic fragments and linked read sequencing. The voucher specimen from which this genome sequence derived is currently stored at −80 °C at the USGS Fort Collins Science Center.

### RAD sequencing

RAD sequencing libraries were generated for 96 samples from each cohort. Genomic DNA was digested with the restriction enzymes Spe1 and Sau3A1. The 20 µL double-digest reaction consisted of 1 µg genomic DNA (13 µL at a concentration of 77 ng/µL), 2 µL 10X T4 DNA Ligase Buffer (New England BioLabs), 1 µL Spe1 (New England BioLabs), 1 µL Sau3A1 (New England BioLabs), 0.2 µL BSA (New England BioLabs), and 2.8 µL water. The digestion was performed at 37 °C for 2 h, after which the solutions were heated to 65 °C for 20 min to denature the enzymes. After reverting the solution temperature to 37 °C, 1 µL of individually-barcoded P2 adaptor and 1 µL of P1 adaptor were added to each sample. To minimize the formation of adaptor-dimers the reactions were allowed to equilibrate to 37 °C before the addition of 1 µL T4 DNA Ligase (New England BioLabs). The adaptors were ligated at 16 °C for 30 min and the temperature was then raised to 65 °C for 20 min to denature the enzymes. The reactions were then brought up to 100 µL with the addition of 80 µL water and then cleaned with 0.65X SpriSelect Beads (Beckman Coulter). The libraries were amplified by PCR in 10 µL reactions consisting of 2 µL cleaned adaptor-ligated DNA, 1 µL 10X AmpliTaq Gold buffer with MgCl_2_, 1 µL dNTP mix, 0.2 µL 10 µM forward primer, 0.2 µL 10 µM reverse primer, 0.2 µL AmpliTaq Gold DNA Polymerase (Life Technologies), and 5.4 µL water. Nine separate PCRs were performed (and subsequently pooled) for each sample using the following protocol: 95 °C for 10 min; 22 cycles of 95 °C for 30 s, 55 °C for 30 s, and 72 °C for 30 s; and a final extension at 72 °C for 5 min. The individual samples were grouped into four pooled libraries of 48 samples each, with each cohort spread across the four pools to mitigate batch effects. Equal volumes of PCR product were pooled for each set of 48 samples, cleaned using 1X SpriSelect beads, and run on a PippinPrep (Sage Science) to select fragments in the size range of 300-500 bp. Each size-selected library pool was run on a BioAnalyzer high sensitivity DNA chip (Agilent Technologies) to validate size and DNA integrity (Fig. S1).

Sequencing was initially performed at the University of Oregon Sequencing Core on the Illumina HiSeq platform to produce 100-nucleotide reads. A second round of sequencing was performed by Genewiz to increase coverage of select libraries. Specifically, we repeated 36 samples in two lanes of 15 and 24 samples, respectively; three of the 36 samples were loaded in both lanes. Due to provider constraints the layout of these latter runs was paired and 150 nucleotides in length. We did not use the second read and trimmed the first read to match the original read length. Fastq sequences are available under National Center for Biotechnology Information (NCBI) BioProject PRJNA604255.

### RADseq read filtering and clustering into tags

RADseq reads were visualized with FASTQC (Andrew, 2020) to confirm the restriction mark and evaluate overall run quality. The initial sequence runs had highly uneven coverage among samples generally and coverage was lower on average in the older cohort. Furthermore, the appended indexes that mark the sample of origin could not be scored for approximately 20% of reads. We were able to recover a fraction of these unassigned reads using cutadapt (Martin, 2011) by assigning index reads that were one edit distance from an expected six-base index to the corresponding sample, provided the edit distance was three or more to all other expected indices. Nonetheless, a substantial fraction of samples was insufficiently represented for analysis and the average coverage of the 2009–2010 cohort was approximately half that of the 2017–2018 cohort. It was to address this coverage deficit that a second sequencing run was ordered for low-coverage samples, as described above, which occurred after the initial clustering of reads into loci.

Reads were modulus trimmed with bbduk (Bushnell, 2020) using a modulus of five. Exogenous 3′ adapter sequences were identified and trimmed with bbduk with *k* set to 15 and *mink* set to 11 (these parameters specify the word sizes used to search for matches within reads and at read edges, respectively). Bases were quality-trimmed with bbduk using a Phred-scaled threshold of 15. Reads were force trimmed with bbduk to a maximum of 100 nucleotides to account for the read length difference between the two sets of runs. Processed reads were first clustered within each sample with vsearch (Rognes et al., 2016) at 95% (*iddef* was set to 1). The resulting tags were then pooled across all samples and reclustered by the same method. Tags found in only one specimen (i.e., a cluster size of one after the reclustering step) were excluded outright.

### Genome assembly and tag filtering

Linked-read assemblies were performed with the supernova assembly program (Weisenfeld et al., 2017) and optimized according to the package guidelines. Multiple iterations of the assembly were performed on the US Geological Survey’s Yeti supercomputer (U.S. Geological Survey, no date) with the final assembly achieving coverage metrics very close to recommended targets for mammals (File S2). Raw fastq and scaffolds for each pseudohaploid assembly are available under NCBI BioProject PRJNA601154. However, because NCBI policy requires contigs or scaffolds to contain no gaps of 15 bp or more (National Center for Biotechnology Information, no date), those assemblies are somewhat more fragmented than the original supernova output, which is available from Cornman et al. (2021). We used the latter in our analysis because the support for scaffolds in high-coverage linked-read data should be robust and longer scaffolds are inherently more useful for our objectives.

Tags were aligned to the genome using BLASTN v. 2.9.0 (Camacho et al., 2009) and those with multiple strong matches were removed, defined as matches with an alignment length greater than 90 bases and summed mismatch and gap scores of less than 15. Tags with no match or a single match that was insufficiently stringent, defined as an alignment length of 95 or less (100 maximum) or a percent identity of 95 or less, were removed as well.

Probable autosomes were identified by assessing coverage patterns for scaffolds greater than 10 Mb in length. We first subsampled up to 2,000,000 reads from each RADseq sample and then mapped these to the reference genome with bowtie2 (Langmead & Salzberg, 2012) using the “end-to-end” and “fast” parameter switches. Read depth at each genomic coordinate was determined with the samtools depth command, excluding zero-coverage sites and subsampling the output 1:50. From these subsampled values, coverage at nonzero sites was averaged for each scaffold and the variance among samples determined. This process identified consistent differences in scaffold coverage attributable to ploidy (see ‘Results’).

The trajectory of Ne in the North American hoary bat over evolutionary time was inferred from the assembled reference genome using the pairwise sequentially Markovian coalescent (psmc) model of Li & Durbin (2011). The psmc software requires a compacted representation of genome heterozygosity as input, in which successive 100-bp windows of the reference genome are converted to one of three state characters: no heterozygous positions occur in the window (“T”), the window contains at least one heterozygous position (“K”), or the window contains one or more ambiguous bases (“N”). The diploid consensus was generated by arbitrarily selecting one of the two pseudohaplotypes produced by the supernova assembly as the reference and converting successive 500-bp windows of each pseudohaplotype into fasta-formatted pseudoreads. The two pseudoread pools were then mapped back to the reference with bowtie2 using the “sensitive” and “end-to-end” parameter switches. Read mappings were retained if they contained fewer than 5% mismatches and ten gap positions and no secondary alignment was reported, which we considered correctly aligned. The consensus sequence of the two alignments was generated with bcftools (Li et al., 2009) after confirming that all variant calls had 1X coverage for both the reference and alternative allele and the called genotype was heterozygous. The 64 intervals over which ancestral Ne can be estimated by psmc were pooled according to the pattern 4 + 25 * 2 + 4 + 6 (see program documentation for details). Confidence intervals were inferred from 100 bootstrap replicates and relative Ne was scaled using a generation time of 2.5 years and a per-base mutation rate of 2.0E−8, which is less than the 2.5E−8 used by Sovic, Carstens & Gibbs (2016) but closer to more recent estimates (Milholland et al., 2017) for human (1.2E−8) and mice (6.0E−9). However, both of those species are phylogenetically distant from bats, for which no independent estimate is available to our knowledge.

### SNP identification and filtering

Reads were mapped to the initial set of candidate tags with bowtie2 using the “end-to-end” and “fast” settings and variants called and subsequently filtered using the samtools suite (Li et al., 2009) as described below. Three samples with fewer than one million mapped reads were removed outright from each cohort, resulting in a maximum sample size of 93 for Nb estimation (the “full dataset” hereafter). Despite the additional sequencing performed for older samples, the median number of mapped reads in the older cohort (4.08 million) was two-thirds that of the newer cohort (5.94 million), which likely impacted the ascertainment of heterozygotes.

Loci identified as multicopy or failing to map to autosomes at the specified criteria were removed. An initial list of candidate SNPs was identified with bcftools using the consensus calling method. Indels were also called in order to subsequently remove SNPs that were within five positions of those sites but were not otherwise used. We also removed SNP loci that lacked both heterozygous and homozygous genotypes and enforced a minimum coverage of at least 5X averaged across all samples, to avoid excessive rates of missing genotypes. We excluded SNPs with less than the maximum quality score assignable by the SNP-calling algorithm (QUAL = 999) and required the base-quality bias statistic (“P_BQB_”) to be greater than 0.0001. SNPs with a minor allele frequency (“MAF”) less than 0.05 in the total data set were also excluded at this stage. Only a single SNP was retained from a given tag to avoid pseudoreplication, resulting in an initial set of 12,915 SNPs (File S3).

In addition to these fixed filtering steps, we tested a range of more stringent MAF and P_BQB_ settings in a combinatorial fashion. This is because there is little prior basis for selecting from a range of reasonable parameter values, and because there is an inherent trade-off between SNP quality and SNP quantity. Therefore, an empirical assessment is needed to determine how sensitive Nb estimates might be to these parameter choices. We therefore generated twenty different iterations of the data set by pairing four values of P_BQB_ (0.0001, 0.001, 0.01, 0.1) with five values of MAF (0.05, 0.075, 0.1, 0.15, 0.2). Other bias metrics important for “shotgun” sequencing (i.e., strand bias, position bias, and mapping quality bias) are not directly applicable to RAD sequencing and were not evaluated.

Following this base filtering, we performed two empirical checks of the overall data set. First, individual observed heterozygosity (H_o_), calculated as the proportion of heterozygous genotypes among all called genotypes, was plotted to confirm that samples converged to similar values with increasing coverage. The H_o_ of individuals should be similar unless the population sample includes highly inbred or admixed individuals. H_o_ was plotted for the most stringent combination of P_BQB_ and MAF thresholds to minimize noise from lower-quality loci. Second, the coverage distribution of all loci was plotted to confirm that it was approximately normal around a single mode, as expected for single-copy autosomal loci. Subsequent analyses were performed with either (1) all samples having at least one million mapped reads (“the full data set”), or (2) after further excluding samples and loci deemed to be outliers by these two empirically derived filters (“the reduced data set”).

### Statistical analysis

Population-genetic statistics were calculated with GenePop 4.7 (Rousset, 2008). Allele frequency spectra were tabulated with the stats function of bcftools. Nb was estimated with 95% confidence intervals for each cohort using NeEstimator v. 2.1 (Do et al., 2014). LD statistics were calculated only among retained autosomal scaffolds and not within them. NeEstimator also implements MAF filtering, but for each cohort separately; we used a minimum MAF of 0.05 for all NeEstimator runs, which necessarily applies after the filtering performed with bcftools for the combined data set.

STRUCTURE (Pritchard, Stephens & Donnelly, 2000) was used to evaluate whether any samples derived from a genetically distinct population. The input data set for this analysis was filtered using the universally applied filters described above but prior to applying any P_BQB_ or MAF threshold. Rather, we randomly subsampled 2% of SNPs to obtain an appropriately sized genotype array for structure analysis (2,449 loci). Monte Carlo Markov Chain simulations were run for 50,000 steps following a burn-in of 5,000 steps and with the cluster parameter K specified from 1 to 5. Five simulations with and without an admixture assumption were performed for each K, with our biological expectation being that individuals are not admixed based on previous work (Korstian, Hale & Williams, 2015; Pylant et al., 2016; Sovic, Carstens & Gibbs, 2016). Pairwise relatedness was estimated within each cohort using Coancestry (Wang, 2011).

## Results

### Reference genome sequencing and ancestral Ne estimation

The *L. cinereus* reference genome assembly was 2.11 Gb, which compares well with the size estimated by C value of 2.37 Gb (Smith, Bickham & Gregory, 2013), and had an average linked-read coverage of 42X (File S2). We obtained 49 total scaffolds greater than 10 Mb, comprising 82.1% of the total scaffold length. Scaffold N50 was 35.1 Mb and scaffold L50 was 17. As hoary bat has a haploid chromosome count of 14 (Baker & Patton, 1967), at least some chromosomes are represented by multiple scaffolds and thus not all cases of pairwise physical linkage can be identified. Autosomal scaffolds were readily distinguished from sex-linked scaffolds by their variance in coverage in the population sample (Fig. S2).

**Figure 1:**
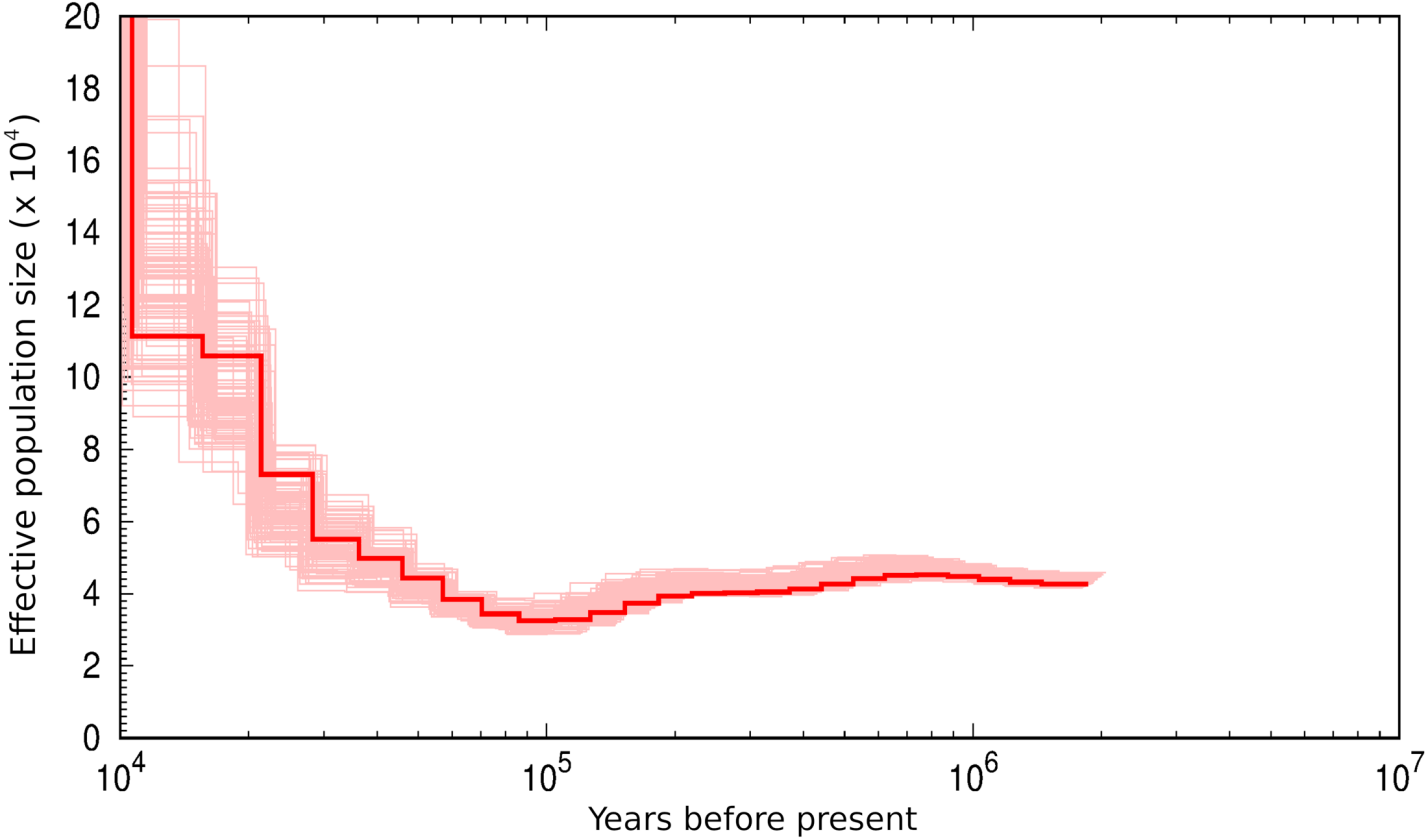
Diploid genome sequence confirms that ancestral effective population size (Ne) of the hoary bat (*Lasiurus cinereus*) has increased over approximately the last 100,000 years. Ancestral Ne was modeled with psmc (Li & Durbin, 2011). The red line represents Ne values estimated from the sequenced genome and pink lines represent bootstrapped confidence intervals (100 iterations of computing Ne from resampled genomic regions). Ne is estimated by psmc for a specified combination of discrete intervals (4 + 25 * 2 + 4 + 6 was used here, see software documentation for details). Only relative Ne can be inferred from coalescence simulations, thus the scaling of the axes requires independent estimates of generation time (g) and mutation rate (µ), which are not known with precision. Here g was set to 2.5 and µwas set to 2.0 × 10E−8.

Analysis of the 45 autosomal scaffolds longer than 10 Mb with psmc indicates that the Ne of the North American hoary bat population at least doubled from ∼100,000 to ∼10,000 years before present (Fig. 1). The trajectory of Ne is robust as indicated by bootstrap resampling (pink lines in Fig. 1), yet the actual magnitude of Ne scales with parameter choices for generation time and particularly mutation rate. Assuming a generation time of 2.5 years and an estimated per-base mutation rate of 2.0E−8 suggests that the Ne of North American hoary bat has exceeded 10,000 in recent evolutionary history. Different values of these parameters would change the axis scaling but not the shape of the curve itself.

### Genetic sexing

Genetic sexing based on sex-linked chromosome dosage (Fig. S2) classified 32 of 93 (34%) total specimens as male in the 2009–2010 cohort and 45 of 93 (48%) male in the 2017–2018 cohort. Fisher’s exact test of the corresponding contingency table revealed no significant association between sex ratio and cohort (*P* = 0.074), but the cumulative number of males is significantly less than 50% (*P* = 0.011 by binomial test) suggesting that wind-turbine mortality may be sex-biased. However, equal sex ratios are not necessarily expected due to sex-based differences in migration and seasonal distribution (Cryan, 2003; Hayes, Cryan & Wunder, 2015). We corroborated the dosage-based sexing method by confirming that the *L. cinereus* ortholog of a known X-linked gene in bats (NCBI accession KC551897 (Korstian et al., 2013)) lies on a scaffold inferred by coverage to be X-linked (Fig. S2). Genetic sexing was performed because carcasses can be difficult to sex morphologically (Korstian et al., 2013), however the sex ratio of each cohort was not expected to influence Nb because only autosomal loci were analyzed.

### SNP filtering and population-genetic comparison of cohorts

Summary statistics for the two cohorts indicate that they derive from very similar pools of genetic variation. The two cohorts also had very similar allele frequency spectra overall (Fig. S3), exhibiting a predominance of low-frequency minor alleles that is consistent with an increasing Ne over recent evolutionary history (Fig. 1 and Sovic, Carstens & Gibbs, 2016). Allele frequencies were not differentiated between cohorts (average *F*_ST_ ≈ 0). The newer cohort had slightly lower diversity than the older cohort based on the Q statistics of Rousset (2008): expected heterozygosity overall (1-Q_inter_) was 0.1802 in the 2017–2018 cohort and 0.1897 in the older cohort. The inbreeding coefficient was somewhat higher in the newer cohort (0.1637) compared with the older (0.1376).

There was an unusually high abundance of C >T transitions, as well as the equivalent (complement) transitions G >A, constituting almost half of all total substitutions (Fig. S4). While this profile is not biologically impossible (Weng et al., 2019), it suggests the possibility that deamination of methylated cytosines and their subsequent conversion to T *in vitro* could be occurring (cf. Briggs et al., 2007; Sawyer et al., 2012), which might accumulate with storage time or correlate with general tissue condition. While overall diversity at C >T sites was similar to that at other sites (1-Q_inter_ of 0.1817 and 0.1876, respectively) and the allele frequency spectra of the two classes of variants were very similar (Fig. S5), out of an abundance of caution we assessed whether Nb differed depending on the variant class used (see below).

Collectively, the STRUCTURE simulations did not suggest the presence of individuals of divergent ancestry in either cohort. Multiple genetic clusters were considered more likely than a single cluster (Fig. S6) only when admixture was assumed (such an assumption is contrary to previous work Korstian, Hale & Williams, 2015; Pylant et al., 2016; Sovic, Carstens & Gibbs, 2016). These additional clusters under admixture were only weakly differentiated from the first and cluster membership was closely related to rates of missing genotypes. Re-running the admixture model after removing samples with missing data rates >10% did not fully eliminate the likelihood advantage of *K* = 2 over *K* = 1, but the second genetic cluster was again weakly differentiated from the first (average *F*_ST_ = 0.096) and constituted a small percentage of total ancestry that was approximately equal across samples and cohorts (Fig. S6). We also note that higher cluster numbers will usually fit the data better, all else being equal, solely due to the higher number of free parameters (Novembre, 2016). We conclude that the two cohorts are comparable and lack substantive genetic admixture, as did Korstian, Hale & Williams (2015), Pylant et al. (2016), and Sovic, Carstens & Gibbs (2016) from independent samples collected in different regions of the continental United States.

For the full data set, the mode value of per-sample coverage (Fig. S7) was approximately 24.8X but deviated from an approximately normal distribution, in that a secondary mode at the lowest coverage values was seen and a long tail of high-coverage loci was also evident. The red lines in Fig. S7 represent subjective demarcations between “expected” and “outlier” coverage; both tails were excluded from the reduced data set, resulting in 6,959 available loci. Plotting H_o_ versus coverage indicates that samples generally converged to similar H_o_ values as coverage increased (Fig. S8), but also reveals outlier samples with either very low or elevated H_o_ (Fig. S8). Low H_o_ was generally associated with low read counts, as expected, but for unknown reasons these samples were classified exclusively as female (Fig. S9).This may be a genuine sex-based difference that remains to be explained or it may indicate that coverage-based sexing fails at low coverage. Samples with elevated H_o_ did not have unusual read coverage but the strongest outliers were all from the older cohort (Fig. S9). The possibility that sample condition or collection protocol contributed to outlier H_o_ in some way is suggested by plotting H_o_ versus sample source (Fig. S10A). Female samples from sample set four had a much wider range of H_o_ than did other sample sets or males from sample set four. The average GC content of sequence reads from outlier samples was often elevated relative to the mode value (Fig. S10B), suggesting a biased loss of AT-rich genomic regions that have lower denaturing temperatures (Mayfield & McKenna, 1978). Based on these results, we only included samples in the reduced data set that had at least 3 million mapped reads and an H_o_ of 0.3–0.4 in the full data set (the blue bounding box in Fig. S8). These empirical thresholds were chosen to mitigate potential outlier effects rather than to definitively categorize ‘good’ versus ‘bad’ samples. The reduced sample sizes under these thresholds were 78 for 2017–2018 and 59 for 2009–2010. Population-genetic statistics of each cohort were more similar for the reduced data set than for the full data set. In the 2017–2018 cohort, 1-Q_inter_ was 0.1830 and *F*_IS_ was 0.1454; in the 2009–2010 cohort, 1-Q_inter_ was 0.1887 and *F*_IS_ was 0.1423. Wang’s coancestry coefficient (Wang, 2002) was less than 0.05 for all pairs in each cohort in the reduced data set, indicating that no closely related individuals were sampled.

### Nb estimates

**Figure 2:**
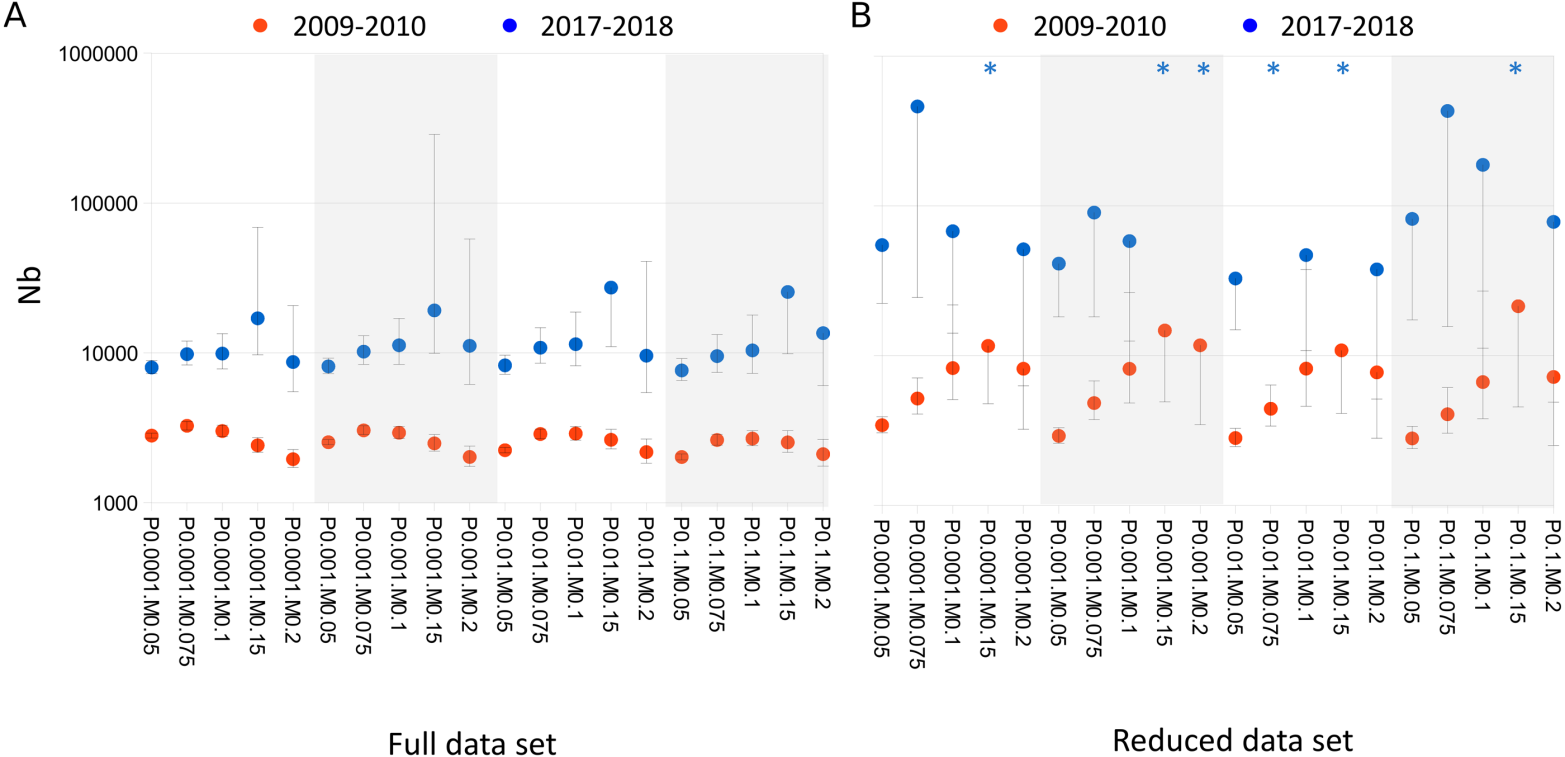
Effective breeding population size (Nb) estimates for the 2017–2018 and 2009–2010 *Lasiurus cinereus* cohorts for tested SNP filtering parameter combinations. (A) Nb is significantly higher for the 2017–2018 cohort for all combinations of minor allele frequency (MAF) and base-quality bias (P_BQB_) for the full data set. (B) Nb is not significantly higher for the 2017–2018 cohort for the reduced data set. Points lacking upper error bars had a 95% confidence maximum of infinity. Asterisks indicate point estimates that were infinite. Horizontal axis labels concatenate P_BQB_ and MAF values, e.g., P0.05M0.1 indicates a P_BQB_ threshold of 0.05 and a MAF threshold of 0.1 were applied.

Nb point estimates fluctuated severalfold across the range of SNP- and sample-filtering strategies investigated (Fig. 2). In all cases, however, Nb was significantly higher in the 2017–2018 cohort than the 2009–2010 cohort when the full data set was analyzed. Nb was more sensitive to MAF, the threshold allele frequency for SNP inclusion, than P_BQB_, the base-quality bias threshold. Point estimates ranged from 7,628 to 27,267 (median 10,281.8) in the 2017–2018 cohort and from 1,950.7 to 3,257 (median 2,574) in the 2009–2010 cohort.

Nb estimates for the reduced data set were again higher in the newer cohort than the older cohort for all filtering combinations, but point estimates and their confidence intervals increased for both cohorts. Point estimates ranged from 32,768.7 to infinite in the 2017–2018 cohort and from 2,801.6 to 21,387.2 (median 7,469.7) in the 2009–2010 cohort. For MAF thresholds of 0.1 or greater, Nb of the old and 2017–2018 cohorts had overlapping confidence intervals regardless of P_BQB_.

**Figure 3:**
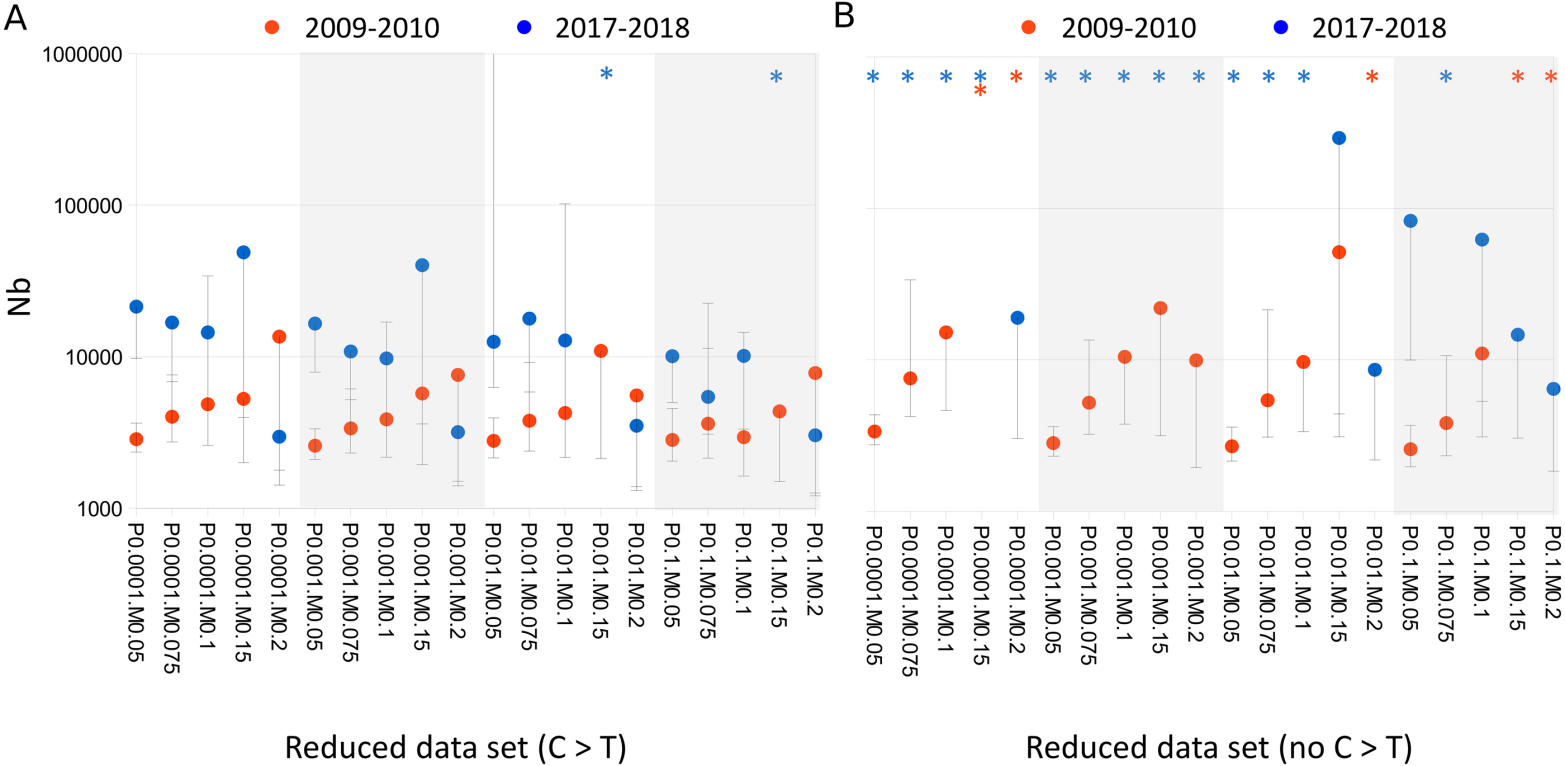
Cytosine to thymine (C > T) variant sites depress effective breeding population size (Nb) estimates relative to other substitution classes. (A) Nb estimates by cohort, considering only cytosine to thymine (C > T) substitutions or their complement, guanine to adenine (G > A). B. Nb estimates by cohort after excluding C > T and G > A substitutions. Points lacking upper error bars had a 95% confidence maximum of infinity. Asterisks indicate point estimates that were infinite. Horizontal axis labels concatenate P_BQB_ and MAF values, e.g., P0.05M0.1 indicates a P_BQB_ threshold of 0.05 and a MAF threshold of 0.1 were applied.

**Figure 4:**
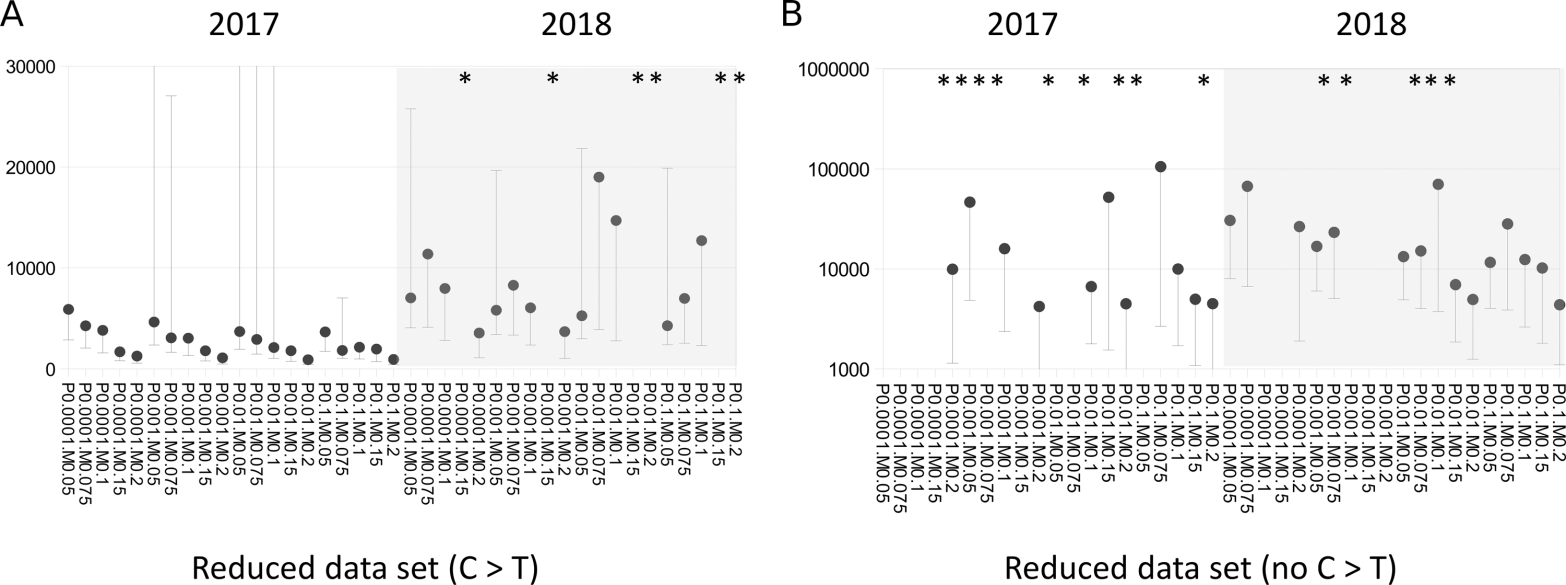
A pronounced year effect on effective breeding population size (Nb) is evident in the 2017–2018 cohort and is alleviated when cytosine to thymine (C > T) sites are excluded. (A) Nb estimates by year within the new cohort, considering only C > T substitutions or their complement, guanine to adenine (G > A). (B) Nb estimates by year within the new cohort, after excluding C > T and G > A substitutions. Points lacking upper error bars had a 95% confidence maximum of infinity. Asterisks indicate point estimates that were infinite. Note the change in axis scale between panels. Horizontal axis labels concatenate P_BQB_ and MAF values, e.g., P0.05M0.1 indicates a P_BQB_ threshold of 0.05 and a MAF threshold of 0.1 were applied.

Stratifying Nb estimates by variant class revealed a strong effect of C >T substitutions on Nb (Fig. 3). Using only C >T substitutions resulted in lower Nb estimates generally than if C >T substitutions were excluded, and tended to recapitulate the values obtained with the full data set. A similar depressive effect of C >T substitutions on Nb could even be seen within the 2017–2018 cohort itself, i.e., by comparing 2017 and 2018 samples (Fig. 4). Samples collected in 2017 yielded much lower Nb estimates than samples collected in 2018 when only C >T sites were used, whereas when C >T sites were excluded, the Nb estimates were similar between the two years.

## Discussion

Temporal trends in contemporary effective population size remain challenging to investigate. Not only are appropriate samples that span the time frame of interest difficult to acquire and potentially of uneven quality, but sampling noise and the lingering influence of past Ne can attenuate temporal signals. In this study, we began with relatively large and balanced cohorts (initial *n* = 96 each) that in all likelihood were separated by more than the generation time of *L. cinereus* (although this cannot be known with certainty). Based on available archives and realized DNA yield, this was the largest cohort size and longest time span that we could feasibly analyze for the North American hoary bat. RADSeq libraries were sequenced on a HiSeq platform in batches of 48 per lane and two additional sequence lanes were used to adaptively supplement low-coverage samples. The average per-sample coverage after initial SNP selection and filtering was ∼25X, which seemed appropriate for our objectives while recognizing that sample coverage remained skewed and was lower on average in the older cohort. While we anticipated higher rates of missing data and allele dropout in the older cohort, which can inflate Nb (Wang, 2016), we initially concluded that frozen carcasses stored on the order of a decade were amenable to high-throughput RADseq analysis. However, this conclusion was undermined by subsequent observations, discussed below.

In addition to the challenge of accurately genotyping archived samples, analytical methods must also have reasonable power to identify changes in Ne or Nb over the time frame of interest. One initial concern was that the LD method may not resolve point estimates when Nb is very large, i.e., above circa 5E+4 based on simulations by Wang (2016), and confidence intervals also increase with the magnitude of the point estimate. It was therefore a potential outcome of this study that Nb of *L. cinereus* would be too high to resolve significant temporal differences. Indeed, *L. cinereus* does appear to have had a large per-generation Ne in recent evolutionary history: at least 1E+4 based on our psmc analysis and up to 1E+5–1E+6 based on the results of (Sovic, Carstens & Gibbs, 2016). In the reduced data set, Nb estimates were frequently infinite or had no upper bound. On the other hand, when estimates were generated by year and with all empirically determined filters imposed (including the exclusion of C >T sites), values were in the vicinity of 1E+4. Thus, it remains possible that incremental improvements in methodology and sampling could resolve *L. cinereus* Nb with sufficient confidence. As they stand, our results are intermediate between the low Nb of 1,546 estimated from a large sample of *L. borealis* (Korstian, Hale & Williams, 2015) and the higher Ne estimates of Sovic, Carstens & Gibbs (2016) for that species and *L. cinereus*.

Overlapping generations are an additional challenge for the LD method, in that the resulting estimates reflect the harmonic mean of per-generation Ne and breeding population Nb over the generations represented (Waples, Antao & Luikart, 2014). At present, the age structure of opportunistically collected hoary bat carcasses cannot be determined from morphological or molecular data, yet the long lifespan of hoary bats allows ample age structure to be present. However, the impact of this uncertainty is likely to be relatively small based on the bias correction proposed by Waples, Antao & Luikart (2014). For example, if we assume a life span of 12 years for hoary bat and an age of first reproduction of 2 years, the bias correction is circa 10%. In comparison, data filtering choices alone altered Nb values by roughly 100% for the full data set. The LD method does have the favorable property of not exhibiting strong lag or bias in response to changing Nb and generally performs well for simulated data (Do et al., 2014; Wang, 2016). Wang (2016) found the sibship frequency method to perform even better than the LD method, but that method is predicated on actually detecting siblings in samples, which appears impractical for hoary bat due to a dearth of relatives in carcass samples (Sovic, Carstens & Gibbs, 2016; Baerwald, Patterson & Barclay, 2014, this study). We therefore believe the LD method remains the best choice for exploring temporal trends in hoary bat effective population size. While various authorities have shown that changes in Nc do not necessarily lead to short-term changes in Nb or Ne (Crandall, Posada & Vasco, 1999), some factors that decouple the two are not likely to be important for *L. cinereus*, such as behavioral inbreeding avoidance that can occur among related individuals in small populations.

### Data filtering choices and their effects

SNP genotypes are inferred from alignment characteristics and are unambiguous when coverage is ample and technical biases are absent. In practice, SNP loci vary greatly in coverage and bias (Andrews et al., 2016), such that some filtering based on quality metrics and allele frequencies is usually necessary, the stringency and character of which depends on study objectives. Filtering steps can be based on general principles that apply per site (e.g., minimum coverage for genotyping, masking gapped regions of an alignment, and requiring genotypic diversity or Hardy-Weinberg equilibrium) and by considering the empirical properties of the data set as a whole (e.g., coverage distributions and inference of ploidy). However, the best filtering combination for a data set is rarely obvious, in part because of tradeoffs between locus number and locus quality. Additionally, factors that may have relatively minor consequences for quantifying population structure, such as allele dropout in heterozygotes, may strongly bias the estimation of LD (Wang, 2016). For this study, we explored filtering parameters implemented in the samtools suite because they are thorough, scriptable, and standardized with the larger body of work on variant detection from sequence alignments (Li et al., 2009).

We investigated a range of P_BQB_ values because per-locus *P*-values are not adjusted for multiple tests and the initial range of values was wide, with the majority of sites having values much lower than 0.01 (Fig. S11). We also examined a range of MAF thresholds, which is an important factor because low-frequency alleles are both more common (Fig. S3) and inherently subject to more false positives. Do et al. (2014) found low-frequency alleles to upwardly bias Nb slightly and Waples & Do (2010) found the precision of Nb over repeated simulations was greatest when MAF was set to 0.1 or above. The latter study also found modest additional benefit of more than 1,000 loci and confidence intervals were estimated too narrowly with increasing marker number. Based on these considerations and given that over 1,500 loci were available in our full data set at the highest stringency (MAF = 0.2, P_BQB_ = 0.1), we would generally have favored the most stringent filtering combination. However, substantially fewer loci were included in the reduced data set, such that intermediate thresholds of MAF and P_BQB_ values may in fact be preferable.

These per-site thresholds indeed had a large effect on Nb estimates, without obvious convergence patterns that might have guided a final selection. The range of point estimates across data filtering choices was particularly striking in the full data set relative to confidence intervals (Fig. 2). Given the observed point-estimate variability, we could not identify a ‘representative’ Nb estimate for each cohort for a given data set. For the full data set, the effects of data filtering were largely parallel between the two cohorts and confidence intervals did not overlap for any parameter combination examined. However, the uncertainty associated with SNP filtering was exacerbated in the reduced data set and other post hoc partitions examined.

The empirically derived bounds we placed on the reduced data set reflected a healthy skepticism of loci with coverage far from the mode value and of samples with H_o_ far from the mean as sequencing effort becomes saturating. We do not argue that all excluded loci and samples are demonstrably ‘bad’ and the remainder demonstrably ‘good’, but we took a prudent approach and examined how Nb was affected by these outlier categories. For example, the long tail of high-coverage loci in Fig. S7 could include loci that appear single-copy in the reference genome assembly but are actually collapsed repeats or segregating copy-number variants, whereas the secondary mode at the lowest coverage values in that figure could represent a higher rate of false positives at low coverage or restriction-site mutations that cause null alleles in some individuals. The clear association of outlier H_o_ with cohort and with sample set implies that systematic differences in condition or storage affected genotype distributions; those effects should be mitigated in the analysis of Nb to the extent possible.

### Impact of substitution type and sample year on Nb

C >T (and its complement, G >A) was the most frequent substitution type, approximately twice as common as the transitions T >C and A >G, and Nb estimates were strongly affected by their inclusion. Apparent C >T mutations can accrue in degraded DNA due to cytosine deamination and subsequent conversion to thymine *in vitro* (Briggs et al., 2007; Sawyer et al., 2012), but C >T mutation rates can also be legitimately high in wild populations (Weng et al., 2019). Furthermore, we lack an independent basis for inferring the ancestral state at these sites, nor could we discern a difference in allele frequency spectrum or heterozygosity at these sites. The Sau3A1 enzyme is methylation sensitive and the methylation state of restriction sites could have changed during storage, but we expect any effect to be random with respect to allelic variation within affected tags. We also found a large difference in Nb between 2017 and 2018 sample years, which appeared to be driving the higher Nb estimates in the newer cohort for the full data set. Since 2010 and 2017 had similar within-year Nb estimates and removal of C >T sites eliminated the among-year difference (Fig. 4), we conclude that no demographic trend can be inferred from the full data set at present.

### Interpreting Nb estimates

Our main interest in estimating hoary bat Nb was as an index of Nc, since the latter measure is presently intractable. That a decline in Nc does not inevitably cause a decline in Ne or Nb has been emphasized (Crandall, Posada & Vasco, 1999), yet the potential of genetic monitoring for this purpose remains strong (Schwartz, Luikart & Waples, 2007; England, Luikart & Waples, 2010; Tallmon et al., 2012; Luikart et al., 2020). The eventual determination of marker recombination rates should expand and strengthen the temporal inferences that can be made from LD (Hollenbeck, Portnoy & Gold, 2016). Evaluations of the scale, stability, and trend in Ne and Nb estimators therefore seem prudent for bat species susceptible to turbine strikes, particularly as carcasses should continue to be available in large numbers*.* Nonetheless, we would cast our analysis as primarily descriptive and not a strong test of the hypothesis of declining Nc, as the variance in Nb attributable to biological or sampling factors remains unknown for *L. cinereus* or related species. Variation in habitat availability, prey availability, weather, or disease are biological factors that could impact the breeding population size in a given season. Interestingly, changes in regional hoary bat occupancy have been documented on a time scale similar to that investigated here (Rodhouse et al., 2019), albeit for an area on the range margin. Those authors found mean occupancy estimates in the northwestern United States to be initially low in the early 2000’s, relatively high circa 2010, and again declining circa 2018, patterns which appeared attributable in part to changing landscape characteristics.

### Ancestral Ne and relevance to Nb

The trajectory of ancestral Ne inferred from the genome of a single *L. cinereus* individual with psmc is on par with the median estimates of contemporary Nb we obtained. For example, assuming the most rigorous Nb estimates derive from the reduced data set, further restricted to 2018 samples and excluding C >T sites (Fig. 4), the median value of 15,144.6 lies in the middle of the range estimated for the most recent historical interval (Fig. 1). However, the scaling of ancestral Ne is sensitive to the mutation rate and generation time assumed. We assumed a slightly lower mutation rate (2.0E−8) than the 2.5E−8 used by Sovic, Carstens & Gibbs (2016) because the former is closer to more recent estimates for humans and mice (Milholland et al., 2017), however the actual mutation rate for *L. cinereus* remains to be determined. While there is no expectation that contemporary Nb and ancestral Ne should closely match, we find the general concordance of the two independent measures reassuring given the wide range of values previously estimated for the genus. Pylant et al. (2016) obtained Ne values most similar to these (6,091, 95% CI [2,481–89,913]), although they did not find significant support for a population growth model versus a stable population model. In contrast, Sovic, Carstens & Gibbs (2016) did find support for a population growth model beginning approximately 150,000 generations ago, yet the magnitude of Ne (estimated at 8.6E+5) and the scale of increase in Ne over that period (roughly 50-fold) obtained by those authors were greater than what is suggested by Fig. 1. A useful follow-up to this genome-based analysis would be to generate comparable estimates from the South American and various disjunct island populations of *L. cinereus*, which might illuminate the timing and demographic consequences of those colonization events.

### Future refinement of the RADseq protocol

The availability of this and other bat genomes (Jebb et al., 2019) allows for further optimization of RADseq methods and for standardizing sampling and data processing across a wide range of species. For example, restriction-enzyme digestion can produce incomparable fragments among individuals if sites frequently differ in methylation state or if a restriction site lies within an active transposable element. Some repeat classes are highly amplified in bats (Pritham & Feschotte, 2007) and are likely variable within and among lineages. *In silico* digestion of reference genomes could be performed after masking repetitive and low-complexity regions to identify enzymes with optimal cut-site distributions with respect to fragment number, fragment size, and consistency across taxa. It may also be useful to compare the quality of genomic extracts by tissue type, in the event that organ tissue is better preserved than the epidermal tissue used here. Considering that relatively large quantities of high-molecular weight genomic DNA are required for repeatable digestion and size selection, shotgun resequencing may ultimately surpass RADseq in efficiency as platform throughput continues to increase.

## Conclusions

The full data set suggests that Nb in the hoary bat cohort collected in 2017–2018 was higher than in the cohort collected in 2009–2010, which is *prima facie* evidence against massive hoary bat population decline due to turbine mortality or any other cause. However, data filtering choices and uncontrollable sample properties had outsized effects on Nb, such that we have little confidence in cohort-level inferences at present. We recommend continued genetic evaluation of opportunistically collected samples, but the utility of the approach will be contingent on an improved understanding of biological and technical sources of variation.

##  Supplemental Information

10.7717/peerj.11285/supp-1 File S1 Sample metadata Collection information for *Lasiurus cinereus* carcasses, including date and anonymized location.

10.7717/peerj.11285/supp-2 File S2 Summary metrics of the analyzed *Lasiurus cinereus* genome assembly reported by the supernova assembly program (Weisenfeld et al., 2017) N50 is the size of the smallest scaffold included in the minimum set of scaffolds needed to contain half the assembly length, whereas L50 is the number of scaffolds in that set. These metrics are commonly used to compare assembly contiguity.

10.7717/peerj.11285/supp-3 File S3 Sample genotype data in variant call format Only single-nucleotide polymorphism sites are included (i.e., indel variants and invariant sites are excluded), after initial filtering as described in the text.

10.7717/peerj.11285/supp-4 Figure S1 Images of fragment-size distributions of final pooled RADseq libraries (48 libraries per pool), produced by an Agilent BioAnalyzer Narrow peaks represent size standards. DQN= DNA quality number, which ranges from one (highly degraded) to ten (highly intact).

10.7717/peerj.11285/supp-5 Figure S2 Genetic sexing of *Lasiurus cinereus* samples by coverage (A) Coefficient of variation (CV) in coverage among samples, for scaffolds greater than 10 MB in length. High CVs indicate different copy numbers among individuals, implying that the scaffolds lie on sex chromosomes. The scaffold to which the sex locus marker of Korstian et al. (2013) aligns is marked. (B) An example of the pattern of scaffold coverage for inferred autosomal versus inferred sex chromosomes. Each point represents relative scaffold coverage in each of the 192 sequenced samples, for two presumed autosomal scaffolds (blue and green dots) and one inferred sex-chromosome scaffold (orange dots). The latter is bimodal in coverage. (C) Ratio of X to autosomal coverage is bimodal in individual samples. Samples with ratios less than 0.65 were considered male. Coverage ratios diverge somewhat from the expected values of 0.5 and 1.0 due under hemizygosity, presumably due to systematic differences in locus ascertainment for a fixed threshold read count.

10.7717/peerj.11285/supp-6 Supplemental Information 6 Allele frequency spectra at variant sites are very similar for the two *Lasiurus cinereus* cohorts Values shown are after filtering single-nucleotide polymorphisms (SNPs) with the base-quality bias threshold (P_BQB_) set to 0.01 and minimum allele frequency threshold (MAF) set to 0.05. Periodic dips are due to the fact that some allele frequencies are less likely solely due to rounding effects.

10.7717/peerj.11285/supp-7 Figure S4 Cytosine to thymine (C > T) substitutions are the most abundant class of single-nucleotide polymorphisms (SNPs) in the data set The first five frequencies approximately mirror the second five frequencies because a given substitution can be expressed two ways depending on which base of each pair of residues is referenced. For example, C > T sites are also G > A sites. Values shown are for the most lenient SNP filtering, i.e. with the minimum allele frequency (MAF) set to 0.05 and the base quality bias (P_BQB_) set to 0.0001.

10.7717/peerj.11285/supp-8 Figure S5 Allele frequency spectra are similar for cytosine to thymine (C > T) sites and for other single-nucleotide polymorphism (SNP) classes Values shown are for the most lenient SNP filtering, i.e. with the minimum allele frequency (MAF) set to 0.05 and the base quality bias (P_BQB_) set to 0.0001.

10.7717/peerj.11285/supp-9 Figure S6 Individual-based evaluation of genetic structure within the two *Lasiurus cinereus* cohorts A. STRUCTURE analysis resulted in highest likelihoods for a single cluster when no admixture was assumed, but more than one genetic cluster under the less plausible assumption of admixture. B. The distribution of ancestry when admixture of two clusters is assumed, for the initial analysis and after removal of samples with missing data rates of 10% or more. Each sample is represented by a bar with the proportion deriving from each cluster colored as indicated. Samples are ordered arbitrarily within each cohort.

10.7717/peerj.11285/supp-10 Figure S7 Distribution of sample coverage of genomic loci containing single-nucleotide polymorphisms (SNPs) Shown are all variable loci passing initial filtering criteria but prior to any filtering by minimum allele frequency (MAF) or base quality bias (P_BQB_). Red lines approximately demarcate inflection points around the mode coverage value of 24.8X. For most analyses, the complete set of loci was used, subject to the MAF and P_BQB_ thresholds, however we also investigated the effect on effective breeding population size (Nb) of excluding loci with outlier coverage, as these may be enriched in technical or biological sources of error.

10.7717/peerj.11285/supp-11 Figure S8 Convergence of observed heterozygosity (Ho) among *Lasiurus cinereus* samples Proportion of called genotypes that are heterozygous converges to approximately H_O_ = 0.3 with increasing coverage, but low and high outliers are evident. Outlier heterozygosity was more frequent at low coverage but also occurred when the total number of mapped reads was high. Ho values shown here were calculated after the most stringent filtering for minor allele frequency (MAF = 0.2) and base-quality bias (P_BQB_ = 0.1) and also after excluding loci with outlier coverage (see Fig. 2). The logarithmic trend line is shown in red and the bounding box around samples included in the reduced data set (see text) is in blue.

10.7717/peerj.11285/supp-12 Figure S9 Biases in observed heterozygosity by sample cohort and sex Samples with unusually high observed heterozygosity (Ho) were predominantly from the older cohort, whereas samples with very low Ho were exclusively classified as female. Ho values shown here were calculated after the most stringent filtering for minor allele frequency (MAF = 0.2) and base-quality bias (P_BQB_ = 0.1) and also after excluding loci with outlier coverage (see Fig. 2).

10.7717/peerj.11285/supp-13 Figure S10 Apparent batch effect of sample set on observed heterozygosity (H_O_) and base composition A. Distribution of H_O_ by sample set (see Supplemental File 1 for sample set groupings). Sample set 4 has a particularly high variance in H_O_, although outliers are not confined to this sample set alone. H_O_ values shown here were calculated after the most stringent filtering for minor allele frequency (MAF = 0.2) and base-quality bias (P_BQB_ = 0.1) and also after excluding loci with outlier coverage (see Fig. 2). B. Proportion of bases that are guanine or cytosine (%GC) in raw sequence reads by sample set. Higher GC is suggestive of DNA degradation due to biased loss of low-GC sequence. Samples with outlier H_O_ also tend to have high GC.

10.7717/peerj.11285/supp-14 Figure S11 Distribution of base-quality bias statistics for all single-nucleotide polymorphisms (SNPs) and samples prior to filtering The red line denotes the cutoff used in filtering the initial data set. Higher (more stringent) thresholds were subsequently evaluated as described in the text.

## Additional Information and Declarations

### DNA Deposition

The following information was supplied regarding the deposition of DNA sequences: The hoary bat genome sequence is available at NCBI: PRJNA601154. The assembly was altered to conform with the conventions of that repository. By agency policy, a separate data release has been created within the ScienceBase repository, which provides the original output of the assembly program. The repository is available at: Cornman RS, Fike JM, Oyler-McCance SJ, Cryan PM. 2021. Genetic variation in hoary bats (Lasiurus cinereus) assessed from archived samples: U.S. Geological Survey data release, https://doi.org/10.5066/P9VSG54Z. The raw RADSeq data and sample metadata is available at NCBI: PRJNA604255.

### Data Availability

The following information was supplied regarding data availability: The hoary bat genome sequence is available at NCBI: PRJNA601154. The raw RADSeq data and sample metadata is available at NCBI: PRJNA604255.
