# Supplementary material for: Historical effective population size of North American hoary bat (Lasiurus cinereus) and challenges to estimating trends in contemporary effective breeding population size from archived samples
Source: PeerJ. 2021 Apr 19;9:e11285. doi: 10.7717/peerj.11285 (PMC8061578; doi:10.7717/peerj.11285)
Supplement: File S2 — N50 is the size of the smallest scaffold included in the minimum set of scaffolds needed to contain half the assembly length, whereas L50 is the number of scaffolds in that set. These metrics are commonly used to compare assembly contiguity. [file peerj-09-11285-s002.docx]

File S2. Summary metrics of the analyzed *Lasiurus cinereus* genome assembly reported by the supernova assembly program (Weisenfeld et al. 2017). N50 is the size of the smallest scaffold included in the minimum set of scaffolds needed to contain half the assembly length, whereas L50 is the number of scaffolds in that set. These metrics are commonly used to compare assembly contiguity.

| Metric | Value |
| --- | --- |
| Input reads | 1.00 x 10^9^ |
| Raw coverage | 60.14 |
| Final effective coverage | 42.32 |
| Mean linked-read length | 50.75 kb |
| Scaffolds > 10 kb | 2,540 |
| Contig N50 | 120.62 kb |
| Scaffold N50 | 35.11 Mb |
| Scaffold L50 | 17 |
| Assembly length | 2.11 Gb |
| Genome size estimated by supernova (this assembly) | 2.51 Gb |
| Genome size estimated by C content (Smith et al. 2013) | 2.37 Gb |
| Estimated repetitive fraction | 6.17% |
| GC content | 42.65% |
