# Supplementary figures and images for: Historical effective population size of North American hoary bat (Lasiurus cinereus) and challenges to estimating trends in contemporary effective breeding population size from archived samples
Source: PeerJ. 2021 Apr 19;9:e11285. doi: 10.7717/peerj.11285 (PMC8061578; doi:10.7717/peerj.11285)

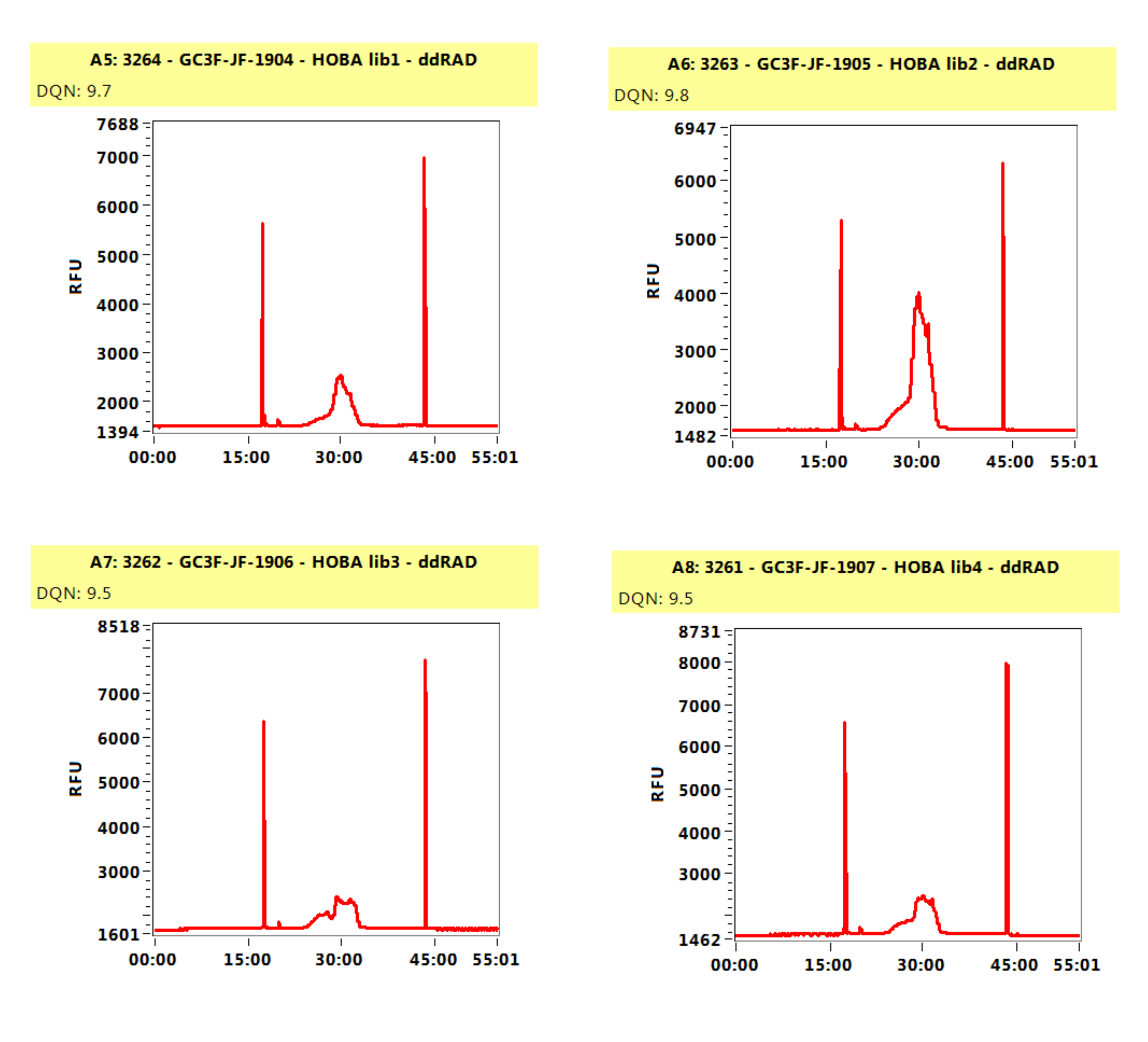

Supplement: Figure S1 — Narrow peaks represent size standards. DQN= DNA quality number, which ranges from one (highly degraded) to ten (highly intact). [file peerj-09-11285-s004.png]

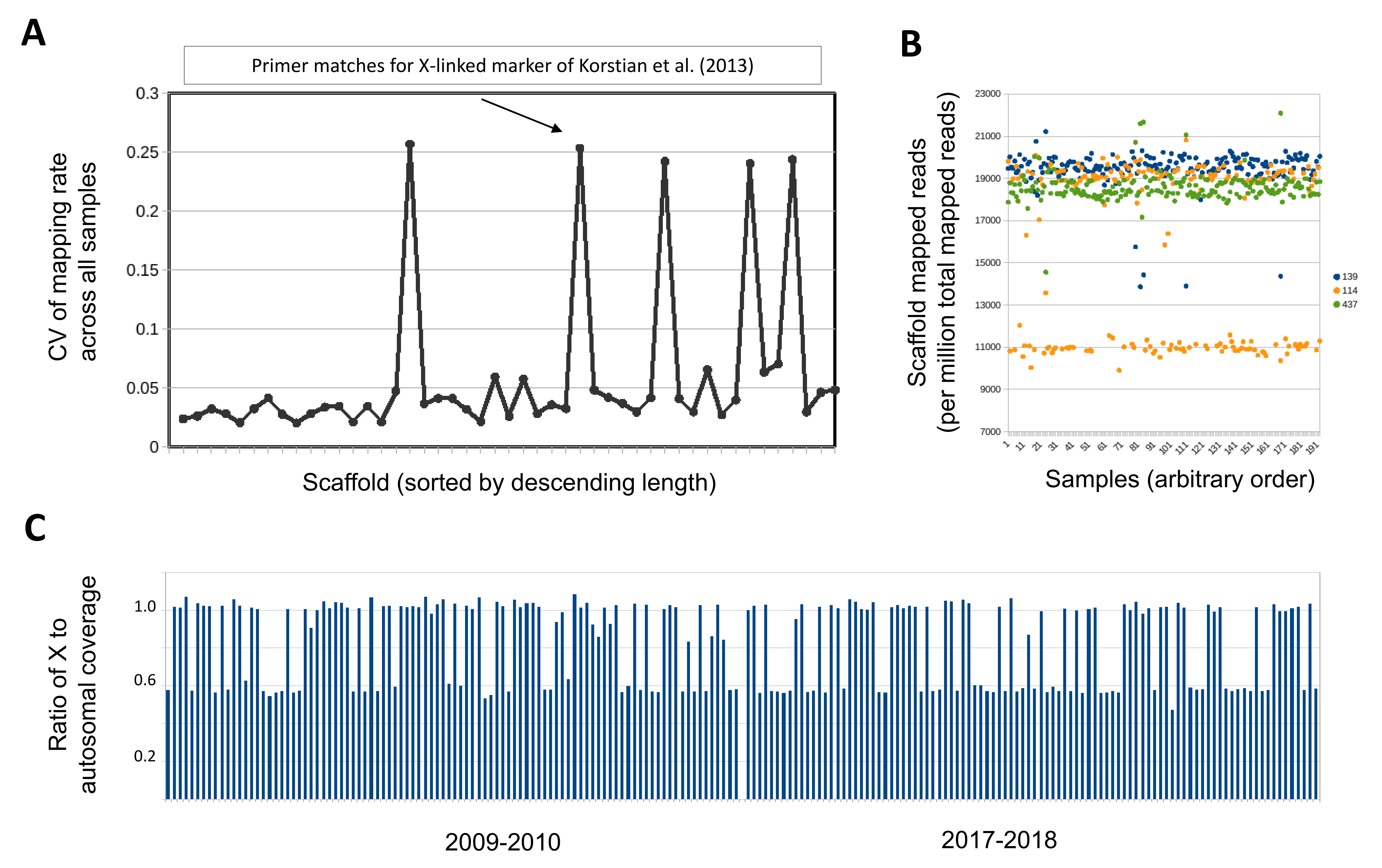

Supplement: Figure S2 — (A) Coefficient of variation (CV) in coverage among samples, for scaffolds greater than 10 MB in length. High CVs indicate different copy numbers among individuals, implying that the scaffolds lie on sex chromosomes. The scaffold to which the sex locus marker of Korstian et al. (2013) aligns is marked. (B) An example of the pattern of scaffold coverage for inferred autosomal versus inferred sex chromosomes. Each point represents relative scaffold coverage in each of the 192 sequenced samples, for two presumed autosomal scaffolds (blue and green dots) and one inferred sex-chromosome scaffold (orange dots). The latter is bimodal in coverage. (C) Ratio of X to autosomal coverage is bimodal in individual samples. Samples with ratios less than 0.65 were considered male. Coverage ratios diverge somewhat from the expected values of 0.5 and 1.0 due under hemizygosity, presumably due to systematic differences in locus ascertainment for a fixed threshold read count. [file peerj-09-11285-s005.png]

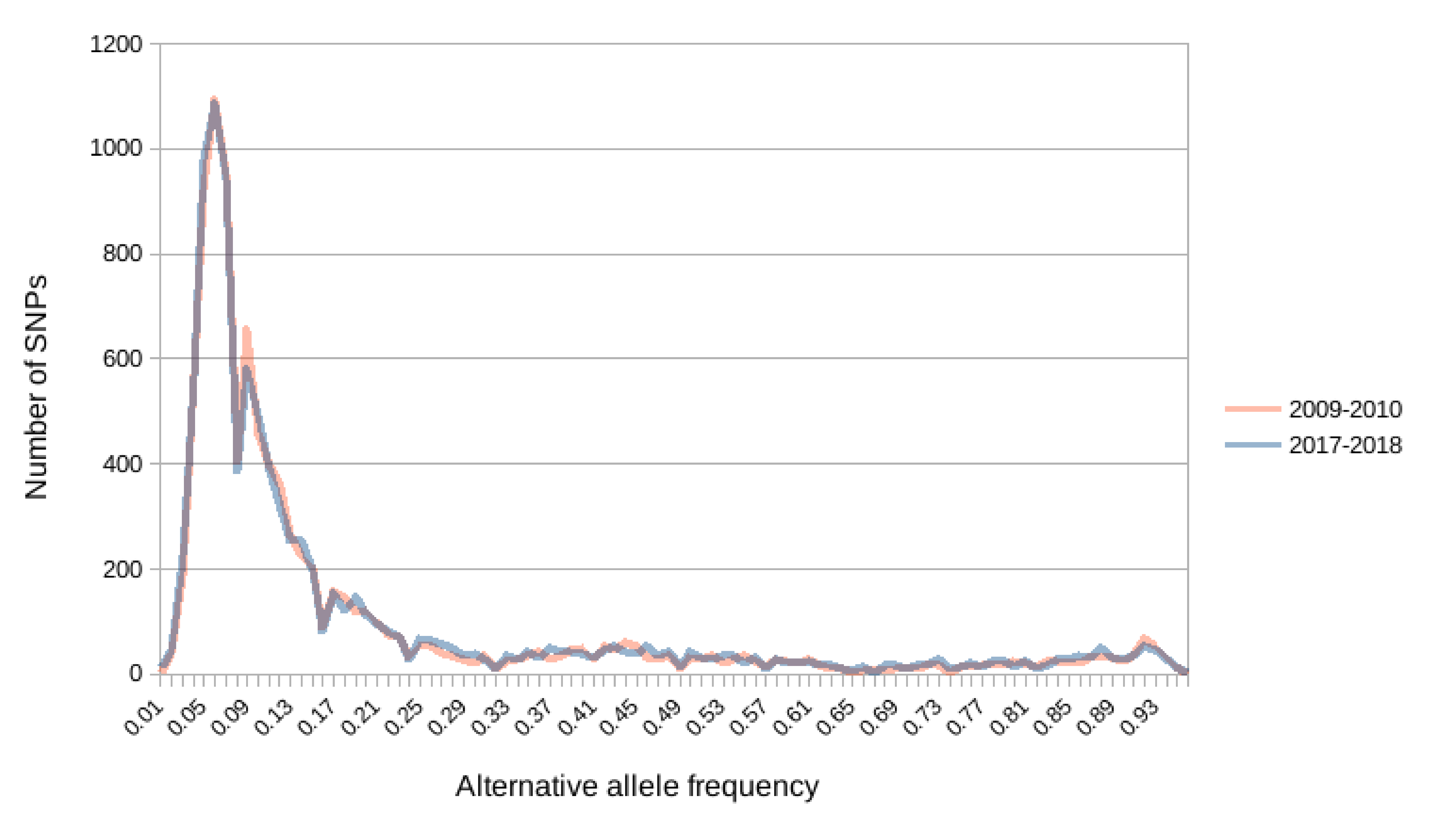

Supplement: Supplemental Information 6 — Values shown are after filtering single-nucleotide polymorphisms (SNPs) with the base-quality bias threshold (PBQB) set to 0.01 and minimum allele frequency threshold (MAF) set to 0.05. Periodic dips are due to the fact that some allele frequencies are less likely solely due to rounding effects. [file peerj-09-11285-s006.png]

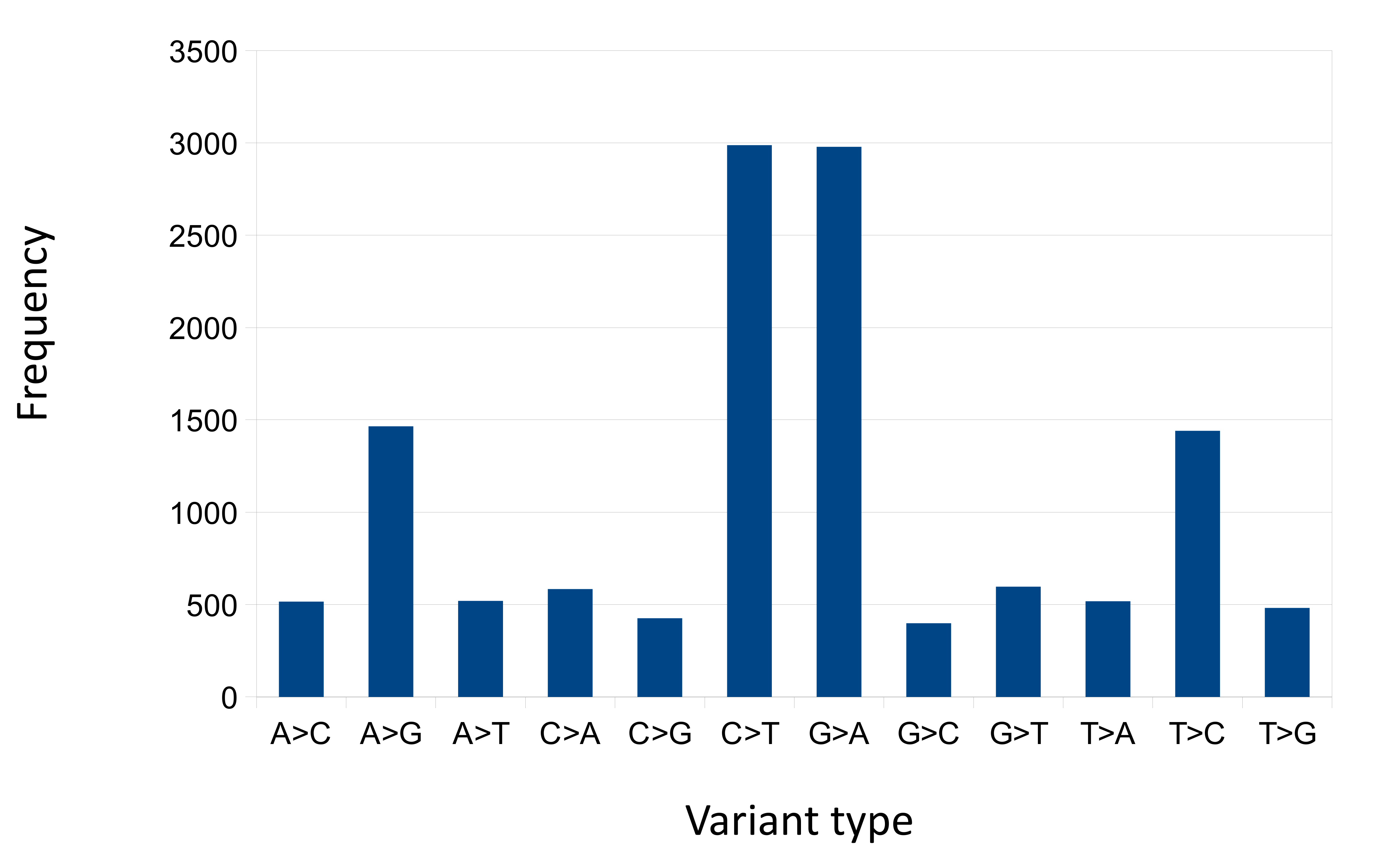

Supplement: Figure S4 — The first five frequencies approximately mirror the second five frequencies because a given substitution can be expressed two ways depending on which base of each pair of residues is referenced. For example, C > T sites are also G > A sites. Values shown are for the most lenient SNP filtering, i.e. with the minimum allele frequency (MAF) set to 0.05 and the base quality bias (PBQB) set to 0.0001. [file peerj-09-11285-s007.png]

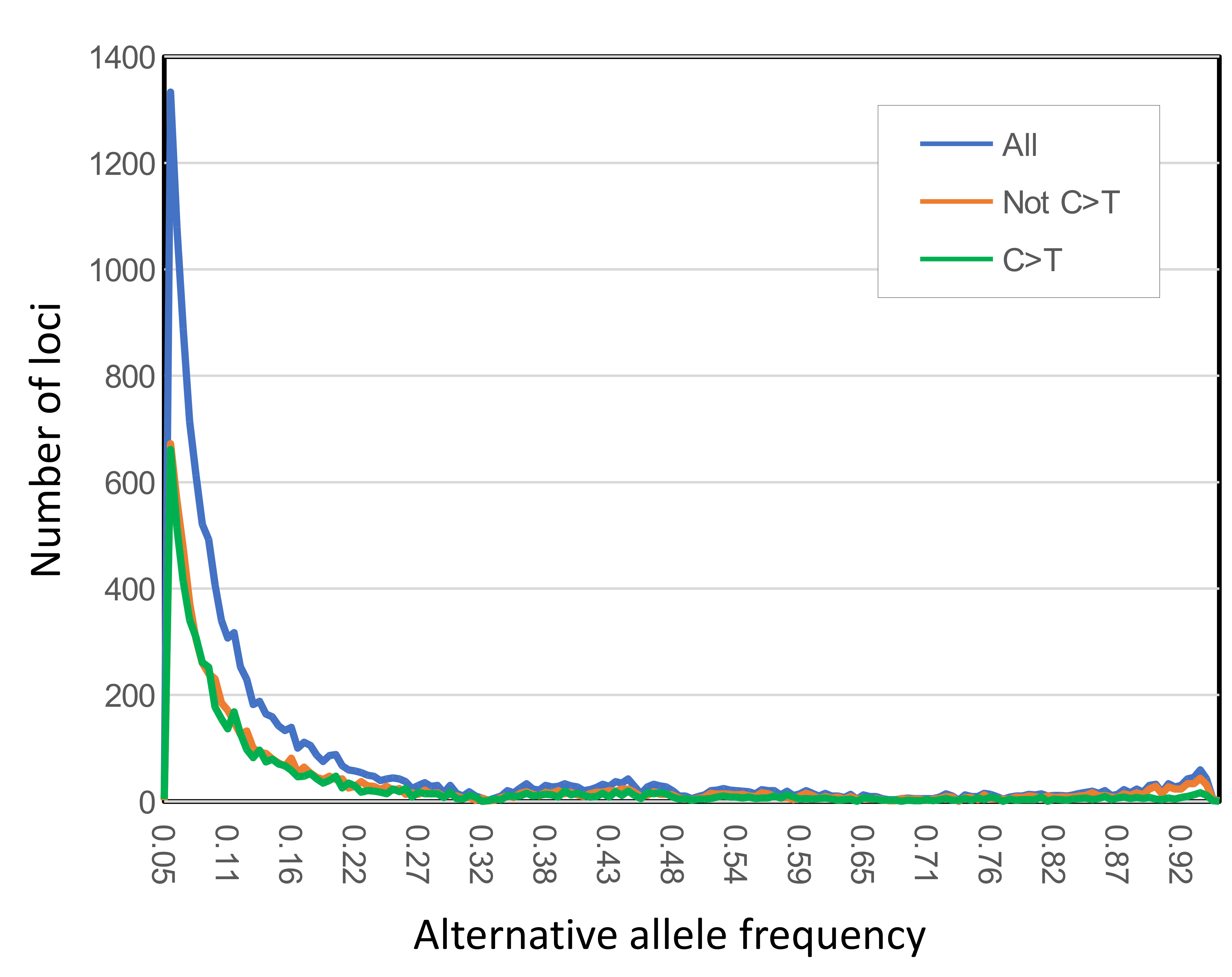

Supplement: Figure S5 — Values shown are for the most lenient SNP filtering, i.e. with the minimum allele frequency (MAF) set to 0.05 and the base quality bias (PBQB) set to 0.0001. [file peerj-09-11285-s008.png]

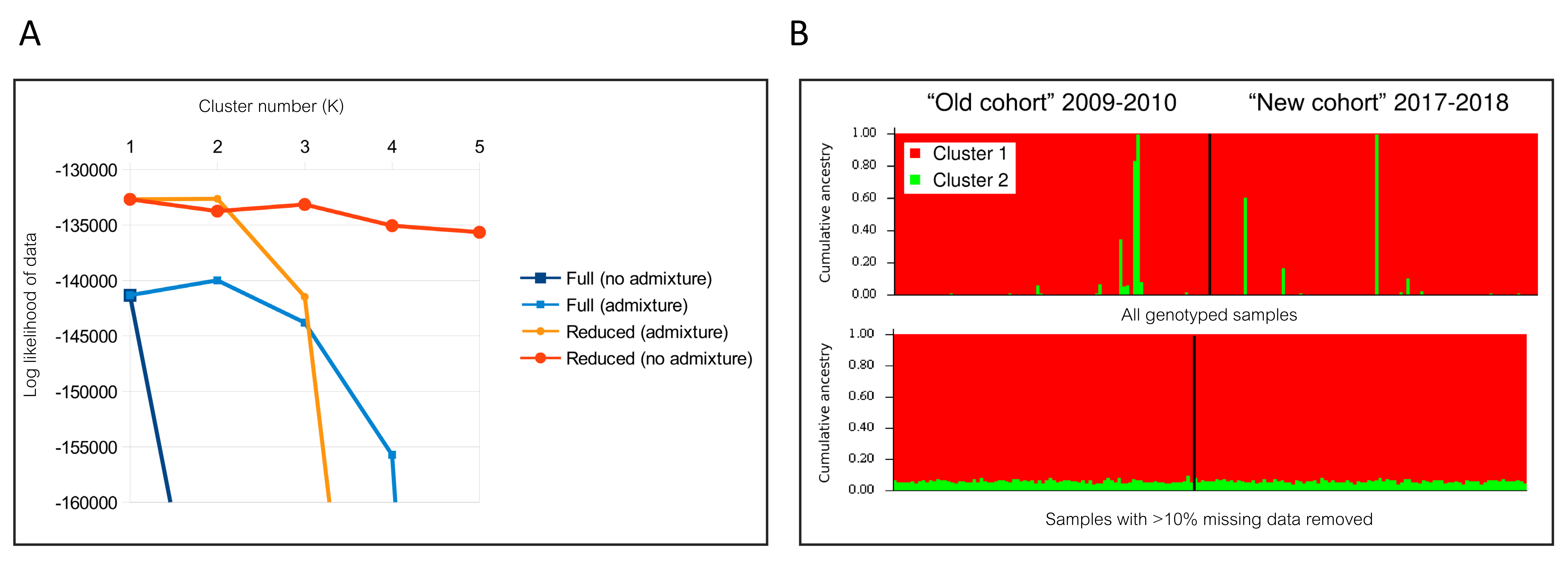

Supplement: Figure S6 — A. STRUCTURE analysis resulted in highest likelihoods for a single cluster when no admixture was assumed, but more than one genetic cluster under the less plausible assumption of admixture. B. The distribution of ancestry when admixture of two clusters is assumed, for the initial analysis and after removal of samples with missing data rates of 10% or more. Each sample is represented by a bar with the proportion deriving from each cluster colored as indicated. Samples are ordered arbitrarily within each cohort. [file peerj-09-11285-s009.png]

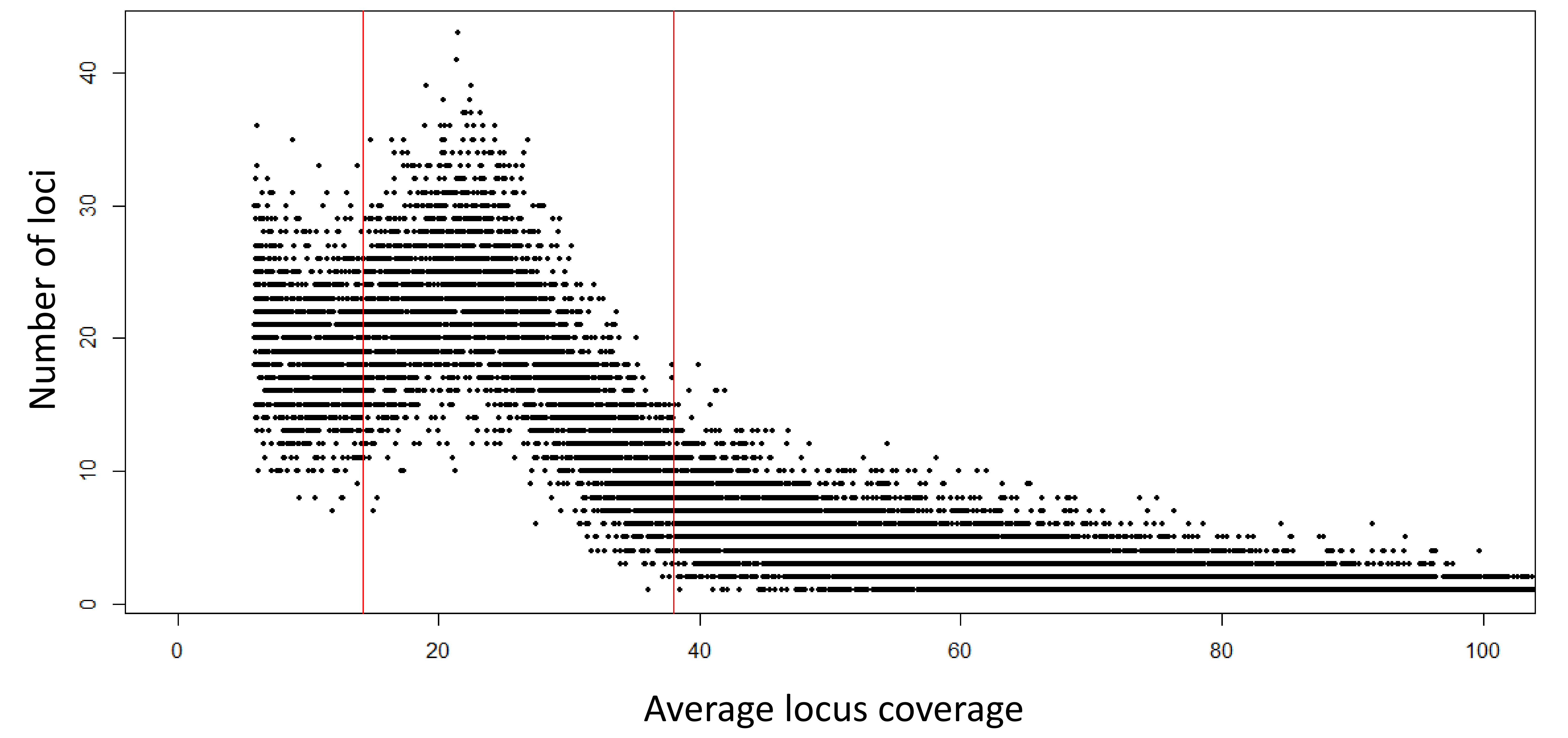

Supplement: Figure S7 — Shown are all variable loci passing initial filtering criteria but prior to any filtering by minimum allele frequency (MAF) or base quality bias (PBQB). Red lines approximately demarcate inflection points around the mode coverage value of 24.8X. For most analyses, the complete set of loci was used, subject to the MAF and PBQB thresholds, however we also investigated the effect on effective breeding population size (Nb) of excluding loci with outlier coverage, as these may be enriched in technical or biological sources of error. [file peerj-09-11285-s010.png]

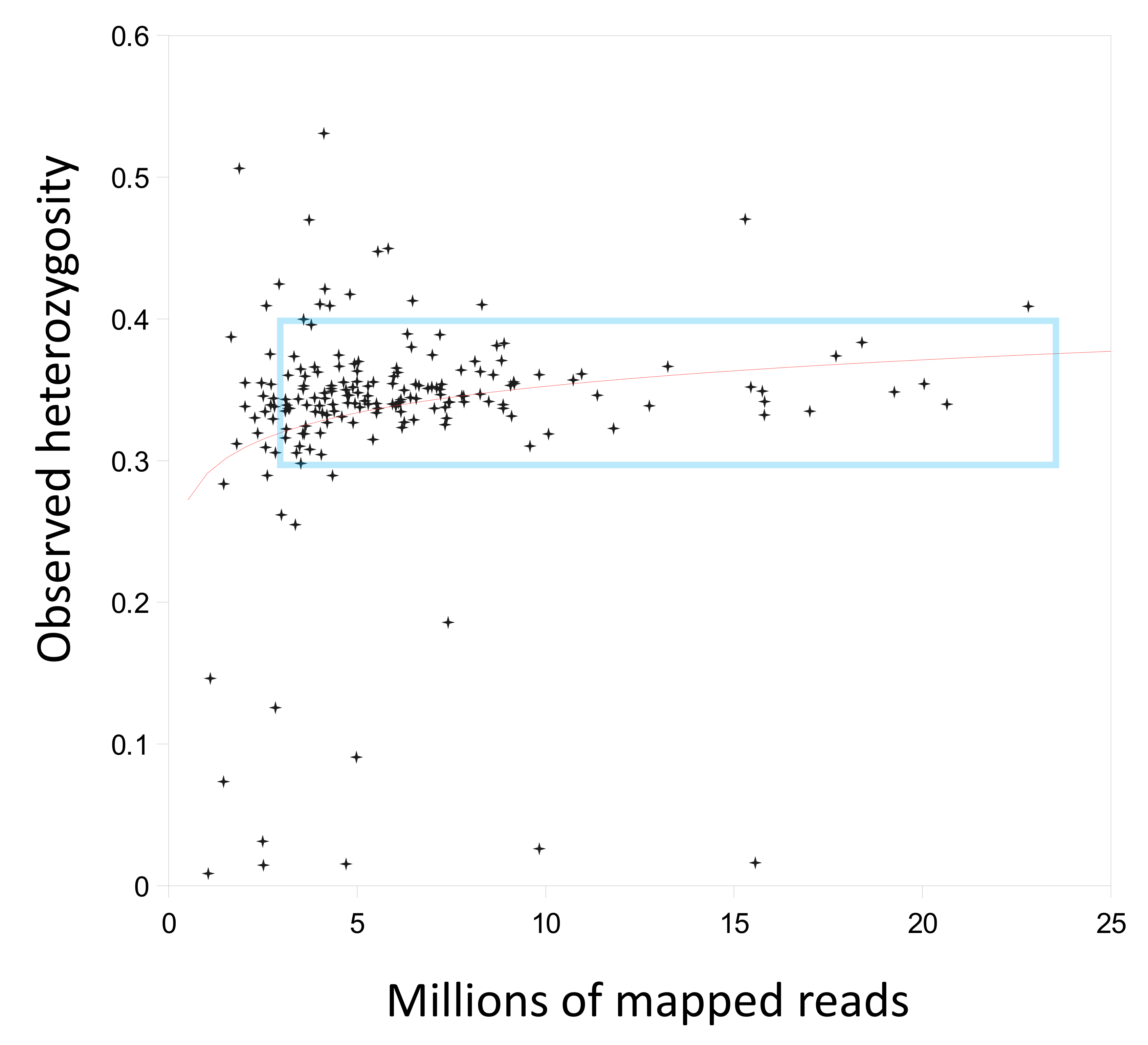

Supplement: Figure S8 — Proportion of called genotypes that are heterozygous converges to approximately HO = 0.3 with increasing coverage, but low and high outliers are evident. Outlier heterozygosity was more frequent at low coverage but also occurred when the total number of mapped reads was high. Ho values shown here were calculated after the most stringent filtering for minor allele frequency (MAF = 0.2) and base-quality bias (PBQB = 0.1) and also after excluding loci with outlier coverage (see Fig. 2). The logarithmic trend line is shown in red and the bounding box around samples included in the reduced data set (see text) is in blue. [file peerj-09-11285-s011.png]

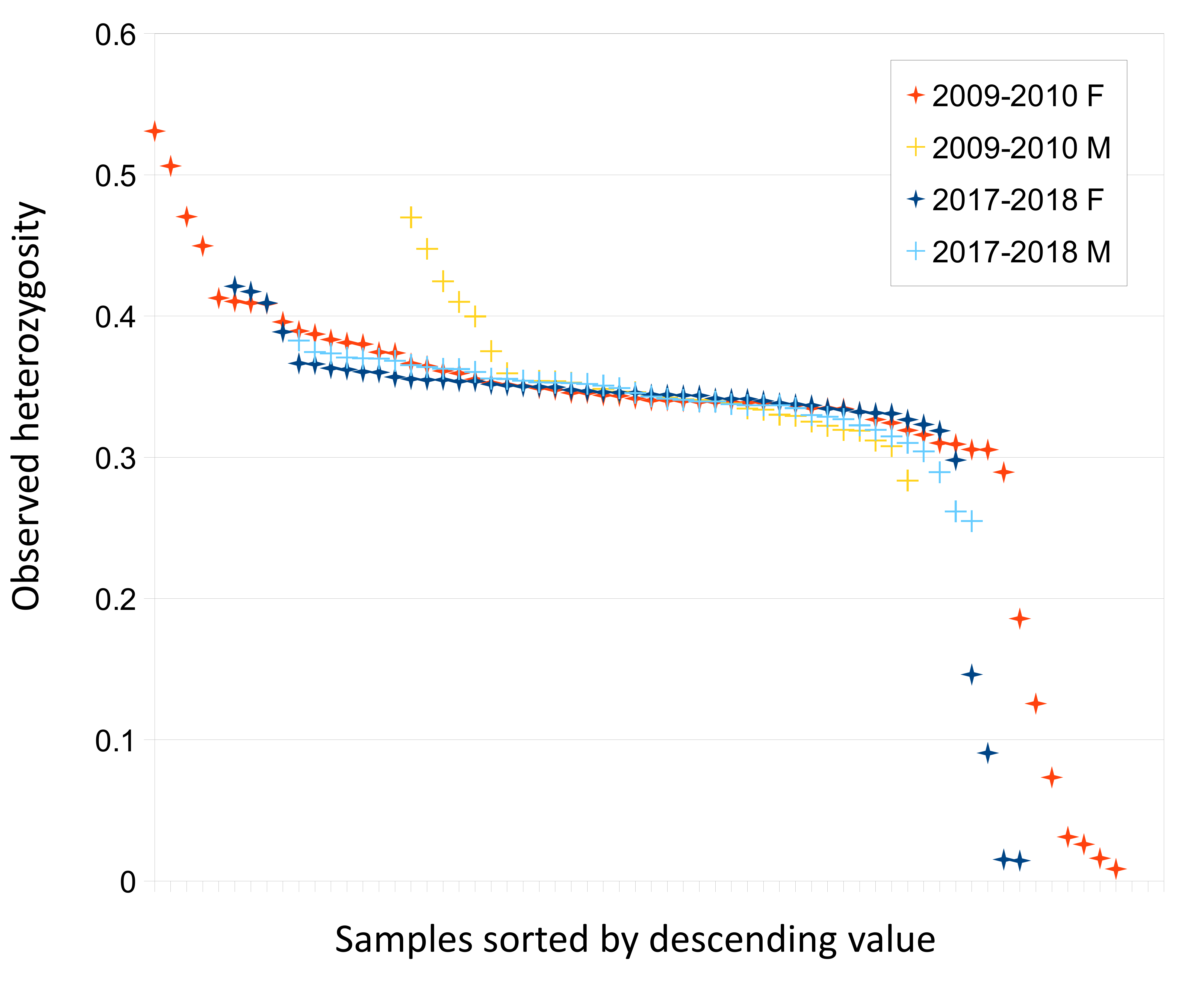

Supplement: Figure S9 — Samples with unusually high observed heterozygosity (Ho) were predominantly from the older cohort, whereas samples with very low Ho were exclusively classified as female. Ho values shown here were calculated after the most stringent filtering for minor allele frequency (MAF = 0.2) and base-quality bias (PBQB = 0.1) and also after excluding loci with outlier coverage (see Fig. 2). [file peerj-09-11285-s012.png]

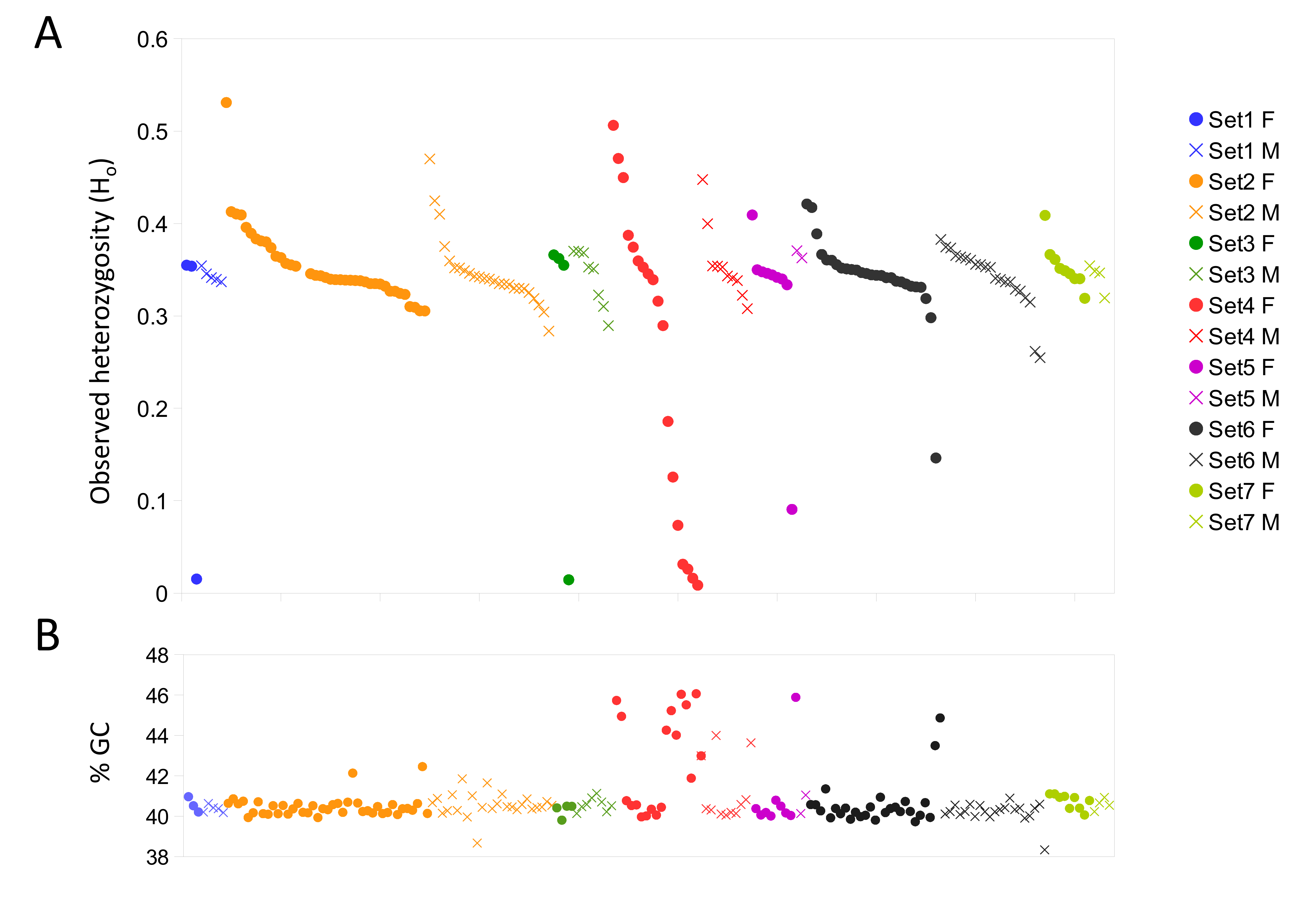

Supplement: Figure S10 — A. Distribution of HO by sample set (see Supplemental File 1 for sample set groupings). Sample set 4 has a particularly high variance in HO, although outliers are not confined to this sample set alone. HO values shown here were calculated after the most stringent filtering for minor allele frequency (MAF = 0.2) and base-quality bias (PBQB = 0.1) and also after excluding loci with outlier coverage (see Fig. 2). B. Proportion of bases that are guanine or cytosine (%GC) in raw sequence reads by sample set. Higher GC is suggestive of DNA degradation due to biased loss of low-GC sequence. Samples with outlier HO also tend to have high GC. [file peerj-09-11285-s013.png]

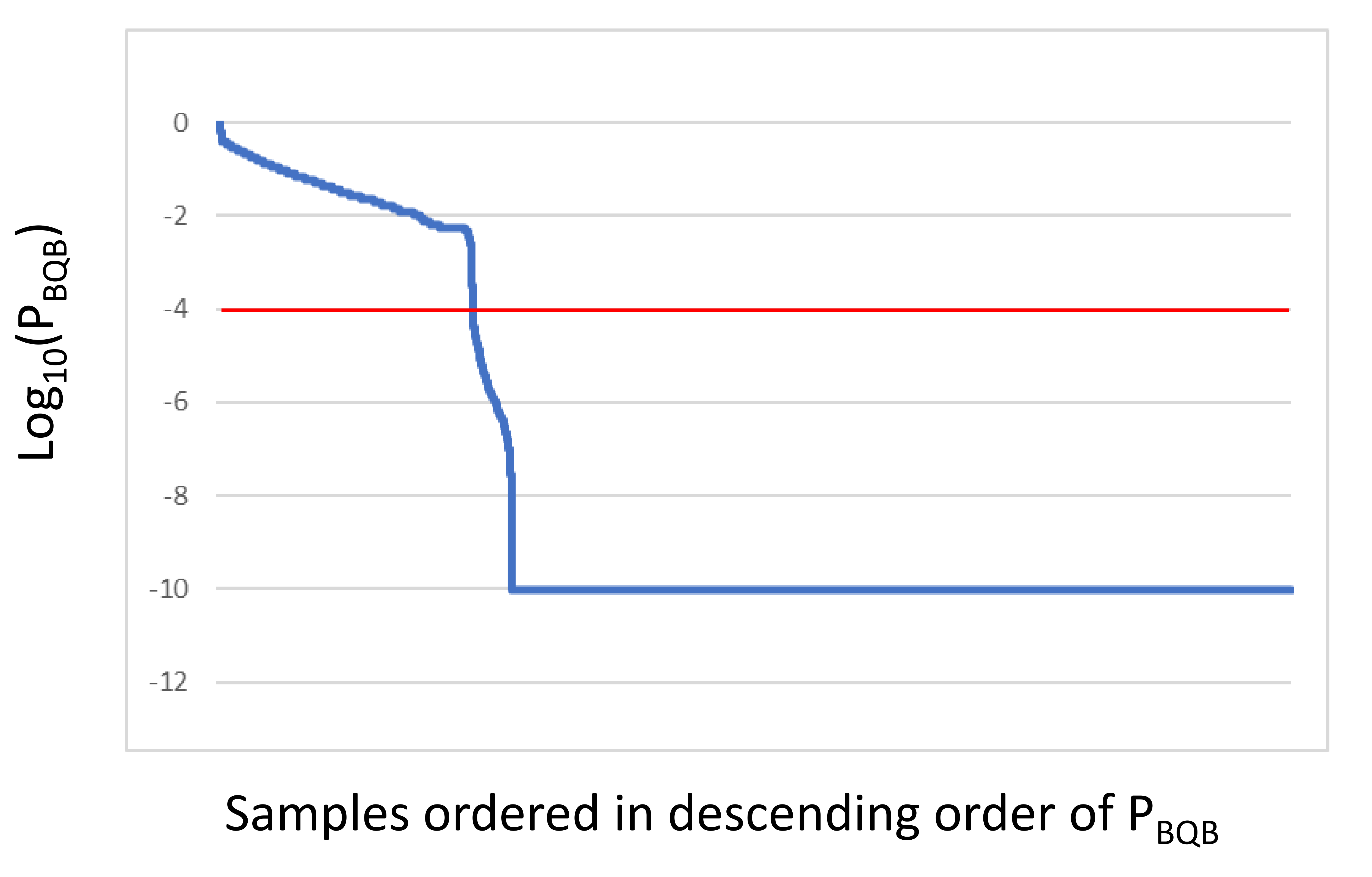

Supplement: Figure S11 — The red line denotes the cutoff used in filtering the initial data set. Higher (more stringent) thresholds were subsequently evaluated as described in the text. [file peerj-09-11285-s014.png]
